# Carbapenemase-producing *Klebsiella oxytoca* complex in France (2017–2023): molecular epidemiology integrated with virulence and biofilm phenotypes

**DOI:** 10.1080/22221751.2026.2713319

**Published:** 2026-08-03

**Authors:** Inès Rezzoug, Océane Vanparis, Joséphine Poiraud, Subidsha Nandakumaran, Delphine Girlich, Léna Latour, Haniel Defoi, Cécile Emeraud, Agnès B. Jousset, Aurelien Birer, Benoit Pilmis, Aymeric Jacquemin, Rémy A. Bonnin, Laurent Dortet

**Affiliations:** a Department of Bacteriology-Hygiene, Bicêtre Hospital, Assistance Publique-Hôpitaux de Paris, Le Kremlin-Bicêtre, France; b Team ‘Resist’ UMR1184 ‘Immunology of Viral, Auto-Immune, Hematological and Bacterial Diseases (IMVA-HB)’, Faculty of Medicine, INSERM, Université Paris-Saclay, CEA, LabEx LERMIT, Le Kremlin-Bicêtre Cedex, France; c French National Reference Center for Antibiotic Resistance, Le Kremlin-Bicêtre, France; d French Associated National Reference Center for Antibiotic Resistance, Bacteriology, CHU Gabriel-Montpied, Clermont-Ferrand, France; e Department of Clinical Microbiology, Saint-Joseph and Marie-Lannelongue Hospitals, Micalis Institute UMR 1319, Université Paris-Saclay, INRAe, AgroParisTech, Paris, France; f SEPSIS Comprehensive Center – IHU SEPSIS

**Keywords:** Carbapenemases, *Klebsiella oxytoca*, epidemiology, Enterobacterales, β-lactamase, virulence, biofilm

## Abstract

**Objectives:**

This study analysed a large French collection of carbapenemase-producing *Klebsiella oxytoca* complex (KoC) isolates (2017–2023) to map species and carbapenemase distributions and identify emerging lineages. We further investigated virulence traits to understand the epidemiological success of the dominant ST-2 lineage.

**Methods:**

We conducted a retrospective study of all KoC isolates received at the French National Reference Center for Antimicrobial Resistance from 2017 to 2023. Whole-genome sequencing was performed to assess species assignment, phylogeny, and sequence types. Virulence analyses included biofilm formation assays (on abiotic surfaces and eukaryotic cells) and *Galleria mellonella* infection models.

**Results:**

*K. oxytoca sensu stricto* remained the most prevalent species among the KoC. OXA-48-like enzymes were the most common carbapenemases, and two major lineages predominated: ST-141 and, far ahead, ST-2. The success of ST-2 appears linked to its low virulence combined with a strong capacity for long-term persistence.

**Conclusion:**

Our findings confirm that *K. oxytoca* comprises a complex of distinct species whose taxonomy warrants continued revision. ST-2 circulates widely in France and, despite its limited virulence, shows remarkable persistence, making it an epidemiological important lineage.

## Introduction

*Klebsiella oxytoca* is a Gram-negative, facultative aero-anaerobic bacillus belonging to the Enterobacterales order. It is a ubiquitous bacterium found in water, soil, and vegetation, and is capable of persisting in healthcare environments [1]. Its high adaptability allows it to survive on inanimate surfaces such as plumbing systems [2] or enteral nutrition flasks [3], resulting in nosocomial outbreaks. Since 1992, at least 27 outbreak-related articles have been published, reporting infections caused by *K. oxytoca* across the globe – from Tunisia to Norway [4,5] and Brazil [6] particularly affecting intensive care units [7].

Beyond outbreaks, *K. oxytoca* can cause sporadic infections, despite being part of the normal gut microbiota [8]. It primarily causes urinary tract infections followed by pneumonia, sepsis, wound infections, and neonatal septicemia [9].

Within the *Klebsiella* genus, *K. oxytoca* ranks as the second most common cause of human infections after *K. pneumoniae* [10]. However, the name *K. oxytoca* often refers to a species complex composed of at least six phylogroups distinguishable by their chromosomally encoded β-lactamase (OXY): *K. oxytoca* (OXY-2), *K. grimontii* (OXY-6), *K. michiganensis* (OXY-1 and OXY-5), *K. spallanzanii* (OXY-3 and OXY-9), *K. pasteurii* (OXY-4), and *K. huaxiensis* (OXY-8) [11] (http://www.bldb.eu/). More recently, OXY-10, OXY-11, and OXY-12 have been described in *K. oxytoca* complex isolates that might belong to three new phylogroups [11]. Differentiation between these species remains challenging using phenotypic methods [11] or MALDI-TOF mass spectrometry, emphasizing the need for genomic sequencing for accurate identification. Among them, *K. michiganensis* and *K. oxytoca* appear to be the most associated with extraintestinal human infections, although the true prevalence of each species remains underestimated, as many isolates reported as *K. oxytoca* may in fact belong to other species within the complex [12].

Members of the KoC are typically susceptible to most of β-lactams, with the OXY enzyme conferring only limited resistance to amino- and carboxy-penicillins. However, over the past decade, ESBL-producing KoC [13] as well as carbapenemases producers have increasingly emerged, mirroring the global rise of carbapenemase-producing Enterobacterales (CPE) [14,15]. The first carbapenem-resistant *K. oxytoca* isolate was described in the United States in 1998 [16]. Since then, a wide diversity of carbapenemases has been identified in the KoC including KPC and GES enzymes (Ambler class A), NDM, VIM, and IMP metallo-β-lactamases (Ambler class B), and OXA-48-like enzymes (Ambler class D) [11,17] with geographical variations in their distribution.

In parallel, the pathogenicity of *K. oxytoca* relies on multiple virulence determinants. It produces cytotoxins such as tilimycin and tilivalline, and, like *K. pneumoniae*, expresses capsular polysaccharides that contribute to immune evasion [18], and adhesins involved in biofilm formation [19,20]. However, the functional role of these genes involved in biofilm production and virulence within the *K. oxytoca* complex remains poorly investigated. Some lineages may exhibit low virulence but high persistence, acting as silent reservoirs, while others may possess higher pathogenic potential but limited epidemiological success [21].

This study aimed to characterize a large collection of carbapenemase-producing KoC isolates collected in France from 2017 to 2023. We first investigated the distribution of carbapenemase types across KoC species and identified potential high-risk lineages. We then focused on the epidemiologically dominant ST-2 lineage to determine whether its widespread dissemination could be associated with enhanced virulence or biofilm-forming capacity.

## Material & methods

### Data collection

A total of 635 *Klebsiella* oxytoca complex clinical isolates collected from 2017 to 2023 at the French National Reference Centre for Antimicrobial Resistance (F-NRC) were included in this study. All isolates had decreased susceptibility to at least one carbapenem (ertapenem, imipenem, or meropenem), according to EUCAST guidelines (https://www.eucast.org/).

Carbapenemase production (or not) was confirmed using the Carba NP test and the NG-Carba5 assay (NG Biotech, Guipry, France), as previously described [22]. For each isolate, information regarding the clinical sample, date of isolation, geographical origin, and specimen type were collected.

This retrospective study focused on carbapenem-resistant Klebsiella oxytoca complex isolates. No patient-identifying information was used; therefore, ethical approval was not required**.**

### Whole-genome sequencing (WGS) and genomic analysis

Whole-genome sequencing was performed on all 635 clinical isolates using the Illumina HiSeq platform (Illumina Inc., San Diego, CA, USA). Sequencing reads were assembled using Shovill v1.1.0 and SPAdes v3.14.0 (https://bio.tools/shovill).

Multi-locus sequence typing (MLST) was conducted using the PubMLST database [23], and resistome analysis was performed with the ResFinder database [24]. The presence of plasmids was investigated by identifying replicon sequences using PlasmidFinder v2.1.

### Phylogenetic analysis and single nucleotide polymorphism-based

A comprehensive phylogenetic analysis was performed, including all 635 isolates. We performed a core-genome-based phylogenetic analysis of our collection. Genome annotation was performed with Prokka v1.14.6, and pan- and core-genome identification was performed with Panaroo v1.5.1 (70% protein identity for pangenome families, 80% presence for core genes). Core genome alignment was performed using MAFFT v7.490. The best-fit model, selected *via* the Bayesian information criterion (BIC) in ModelFinder Plus, guided phylogenetic inference with IQ-TREE v2.0.7. The tree, along with associated metadata, was visualized and annotated using iTOL v6.5.2 (European Molecular Biology Laboratory).

We represented MLST data as a Minimum Spanning Tree using the geoBurst algorithm available via the PHYLOViZ 2.0 project.

In addition, a single-nucleotide polymorphism (SNP)-based analysis was performed on the two main sequence types (STs) identified in this study: ST2 and ST141. For each ST, the following strains have been used as reference genomes: strain CNR20220327 for ST2 and 158A5 for ST141. NGS reads from each group were mapped to their corresponding reference genome using Snippy v4.6.0, and core SNPs were extracted to infer phylogenetic relationships. ST-specific trees were constructed and visualized using FigTree v1.4.4.

### Strains from long-term carriers and genomic relatedness

From the F-NRC collection, we selected six *Klebsiella oxytoca* isolates recovered from three patients with documented long-term intestinal carriage (>6 months; two isolates per patient). All six isolates produced the OXA-48 carbapenemase. For each patient, the two strains within-patient isolates were compared by whole-genome sequencing to assess persistence/relatedness using pairwise core-genome SNP distances. For phenotypic comparisons, five additional OXA-48–producing *K. oxytoca* isolates representing distinct sequence types were included.

### Adhesion/biofilm on polystyrene (96-well plate)

Adherence to abiotic surface was quantified in flat-bottom 96-well polystyrene plates. Wells were pre-treated overnight with 100 μL of poly-L-lysine (Day 0). Overnight Brain Heart Infusion (BHI) cultures were diluted 1:10 into fresh BHI and incubated at 37 °C (200 µL per well, 8 technical replicates per strain). After 24 h incubation, wells were washed three times with PBS, stained with 0.5% crystal violet (20 min), rinsed, and destained with 30% acetic acid. The eluate was transferred to a new plate and absorbance was measured at 570 nm after blank subtraction. PBS-only wells served as negative controls, and *K. pneumoniae* LM21 strain was used a positive control [25].

### Adhesion to HeLa cells

Adhesion to epithelial cells was measured using HeLa monolayers grown in 12-well plates. Bacteria were cultured overnight in BHI, sub-cultured 1:50 into fresh BHI, and harvested at mid-log phase (OD₆₀₀ ≈ 0.8). Pellets were washed three times with DMEM (Dulbecco's Modified Eagle Medium) and adjusted to a multiplicity of infection (MOI) of 20 in DMEM. HeLa monolayers were infected with 1 mL inoculum per well, centrifuged (1,500 g, 5 min) and incubated for 1 h at 37 °C. After five PBS washes, cells were lysed with 100 µL sterile water and scraped; lysates were serially diluted and plated on TSA (Trypticase soy broth) to enumerate cell-associated CFU after overnight incubation at 37 °C. The HeLa cell adhesion assay was designed to evaluate relative differences in adhesion among the *Klebsiella oxytoca* complex isolates included in this study. Since all isolates were tested simultaneously under standardized conditions, no positive or negative control was included for this assay.

### Virulence assay in *Galleria mellonella*

Virulence was assessed in *Galleria mellonella* larvae as described previously [26]. Briefly, overnight BHI cultures were adjusted by serial dilution, and 10 µL were injected into the last left proleg (40 larvae per strain and condition). Larvae were incubated at 37 °C and monitored for up to 99 h for survival and phenotypic changes. For each strain, LT₅₀ (time to 50% mortality) and/or LD₅₀ were determined. A negative-control group injected with PBS was included in every experiment. For each experiment, a control group of larvae injected with PBS was included to account for manipulation-related mortality.

## Results

### Whole-genome phylogeny analysis

**Figure 1.**
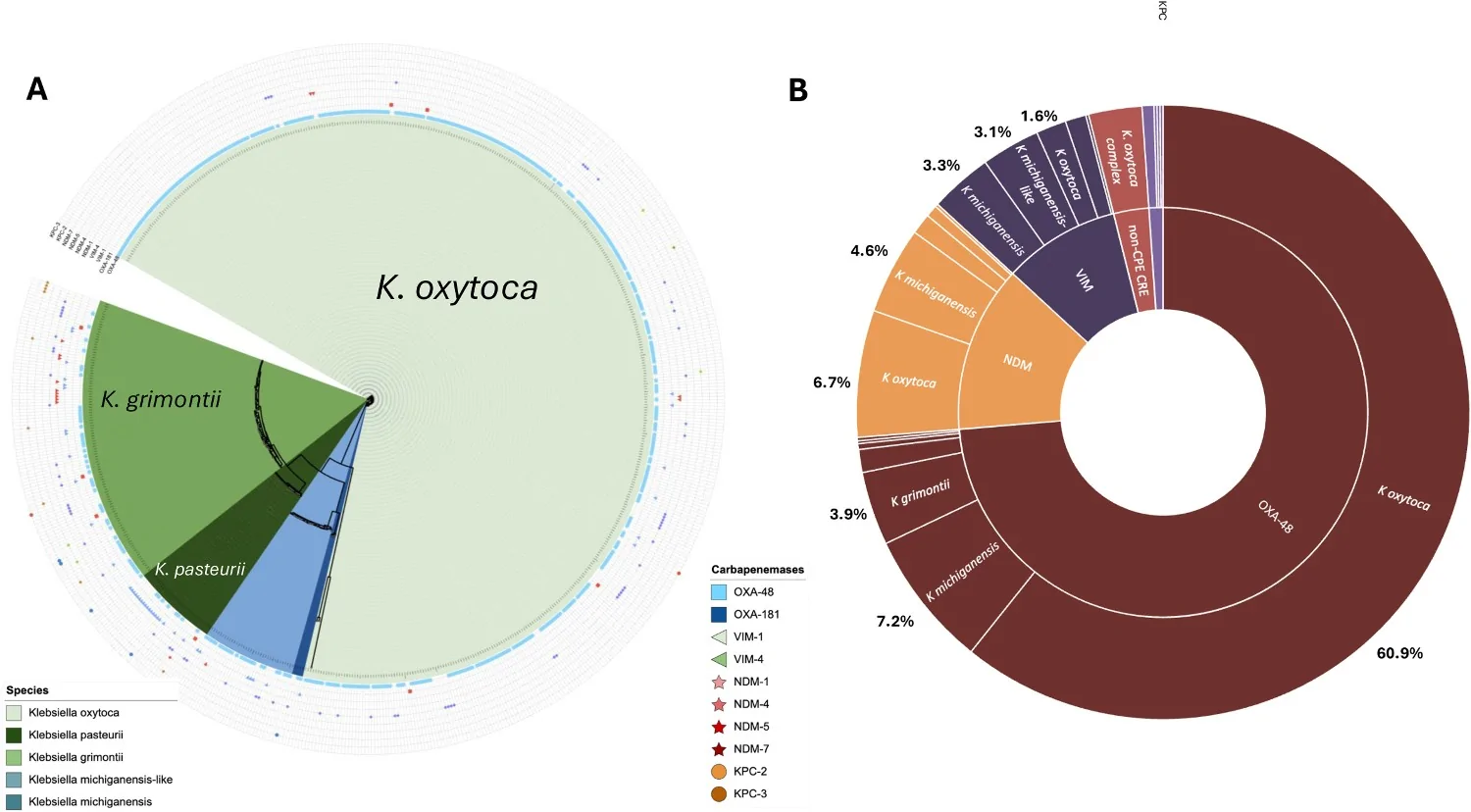
Genetic diversity of carbapenemase-producing *Klebsiella oxytoca* complex (KoC) isolates, 2017–2023 **A**. Phylogenetic tree of carbapenemase-producing *Klebsiella oxytoca* complex isolates collected between 2017 and 2023. Each colour within the shaded clades represents a distinct species of the complex: *K. oxytoca* (bright green), *K. grimontii* (medium green), *K. pasteurii* (dark green), *K. michiganensis* (dark blue), and *K. michiganensis-like* (light blue). Coloured symbols on the outer ring indicate the type of carbapenemase gene detected. **B**. The inner ring represents carbapenemase families (OXA-48–like, NDM, VIM, KPC, IMP), while the outer ring indicates the corresponding species within the KoC. Segment size is proportional to the number of isolates.

Across the 21,215 carbapenemase-producing Enterobacterales (CPE) referred to the F-NRC between 2017 and 2023, the *Klebsiella oxytoca* complex (KoC) represented 3,0% (n = 635). Seven KoC species were identified including *K. oxytoca* the most prevalent (n = 459) – followed by *K. michiganensis* (n = 106), *K. michiganensis-like* (n = 32), *K. grimontii* (n = 31), and less frequently *K. pasteurii* (n = 4) (Figure 1A, Figure S1). Two additional species, *K. spallanzanii* (n = 2) and *K. huaxiensis* (n = 1), were also identified but are not represented in Figure 1A because they are phylogenetically distant from the other KoC species included in the tree.

Overall, OXA-48-like enzymes predominated (475/635; 74.8%), followed by NDM (76/635; 12.0%) and VIM (48/635; 7.6%), while KPC (7/635; 1.1%) and producers were less frequent. The remaining isolates comprised 11 double carbapenemase producers, including 2 NDM/OXA-48-like, 6 VIM/OXA-48-like, and 3 NDM/VIM co-producers (11/635; 1.7%), as well as 18 carbapenem-resistant non-carbapenemase-producing Enterobacterales (non-CPE CRE) (18/635; 2.8%) (Figure 1A-B). Among all isolates, 46.7% (297/635) carried an ESBL, mainly of the CTX-M family, but also SHV- and TEM-type ESBLs, whereas 7.2% (46/635) harboured an acquired AmpC β-lactamase (Table S1). Carbapenemase distribution varied across species. Among OXA-48 producers, *K. oxytoca* accounted for 82.5% of isolates, followed by *K. michiganensis* (9.7%), *K. grimontii* (5.3%), *K. michiganensis-like* (1.7%), *K. pasteurii* (0.4%), and *K. huaxiensis* and *K. spallanzanii* (0.2% each). Among NDM producers, *K. oxytoca* remained most frequent (51.2%), followed by *K. michiganensis* (34.5%), *K. michiganensis-like* (8.3%), *K. grimontii* (4.7%) and *K. pasteurii* (1.2%). VIM producers were mainly found in *K. michiganensis* (35.6%) and *K. michiganensis*-like isolates (33.9%). The remaining VIM producers comprised *K. oxytoca* (16.9%), *K. grimontii* (11.9%), and *K. spallanzanii* (1.7%). KPC producers (n = 7) occurred mainly in *K. michiganensis* (4/7; 57.1%), with one isolate each in *K. michiganensis-like*, *K. oxytoca* and *K. pasteurii*; GES producers were detected predominantly in *K. oxytoca* with additional isolates in *K. grimontii* and *K. michiganensis-like* (Figure 1).

### Sequence type distribution and clonal structure of *Klebsiella oxytoca* complex

To better understand the population structure and dissemination dynamics within the *K. oxytoca* complex, we analyzed the distribution of sequence types (STs) (Figure S1) and their association with specific carbapenemase families.

Among VIM-producing *K. michiganensis*, no single ST predominated, and carbapenemase families were broadly distributed without a discernible phylogenetic cluster, suggesting multiple independent acquisition events. By contrast, among *K. michiganensis*-like isolates, a compact cluster of 16 closely related ST-310 isolates was identified, all producing VIM-1, four of which co-produced OXA-48. All these isolates originated from the Auvergne–Rhône–Alpes region, and SNP analysis revealed 5 - 84 single nucleotide differences, indicating a possible regional dissemination event (supplementary Figure S2).

**Figure 2.**
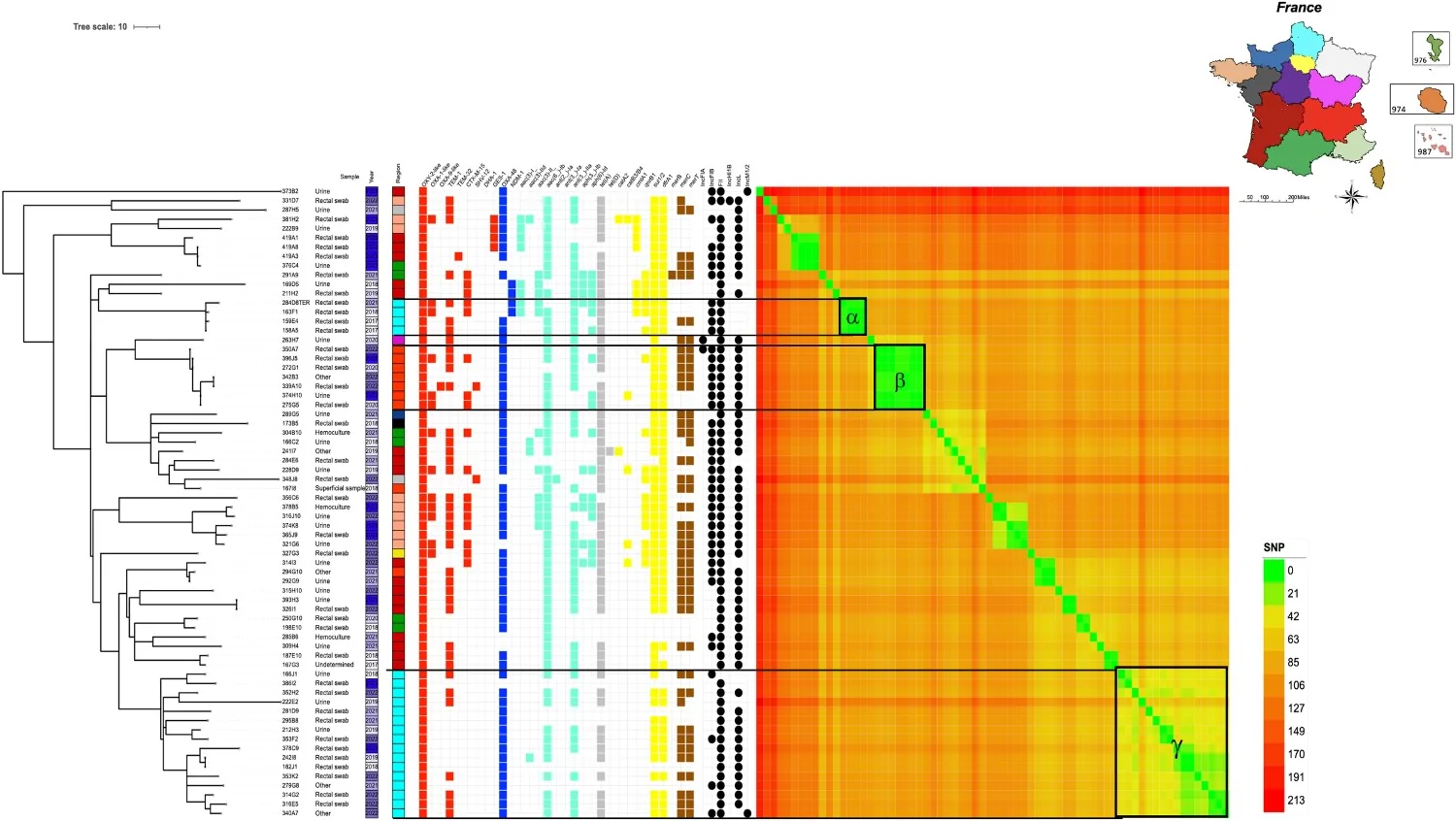
Phylogenic relationships and genomic features of *K. oxytoca* isolates belonging to ST-141. From left to right: phylogenetic tree, isolation site, year of collection, and geographical origin, followed by the presence/absence of antimicrobial resistance genes, virulence determinants, and plasmid replicase types. The right panel displays the pairwise SNP-distance matrix, where colour intensity reflects genetic relatedness between isolates (green = closely related, red = distant).

**Figure 3.**
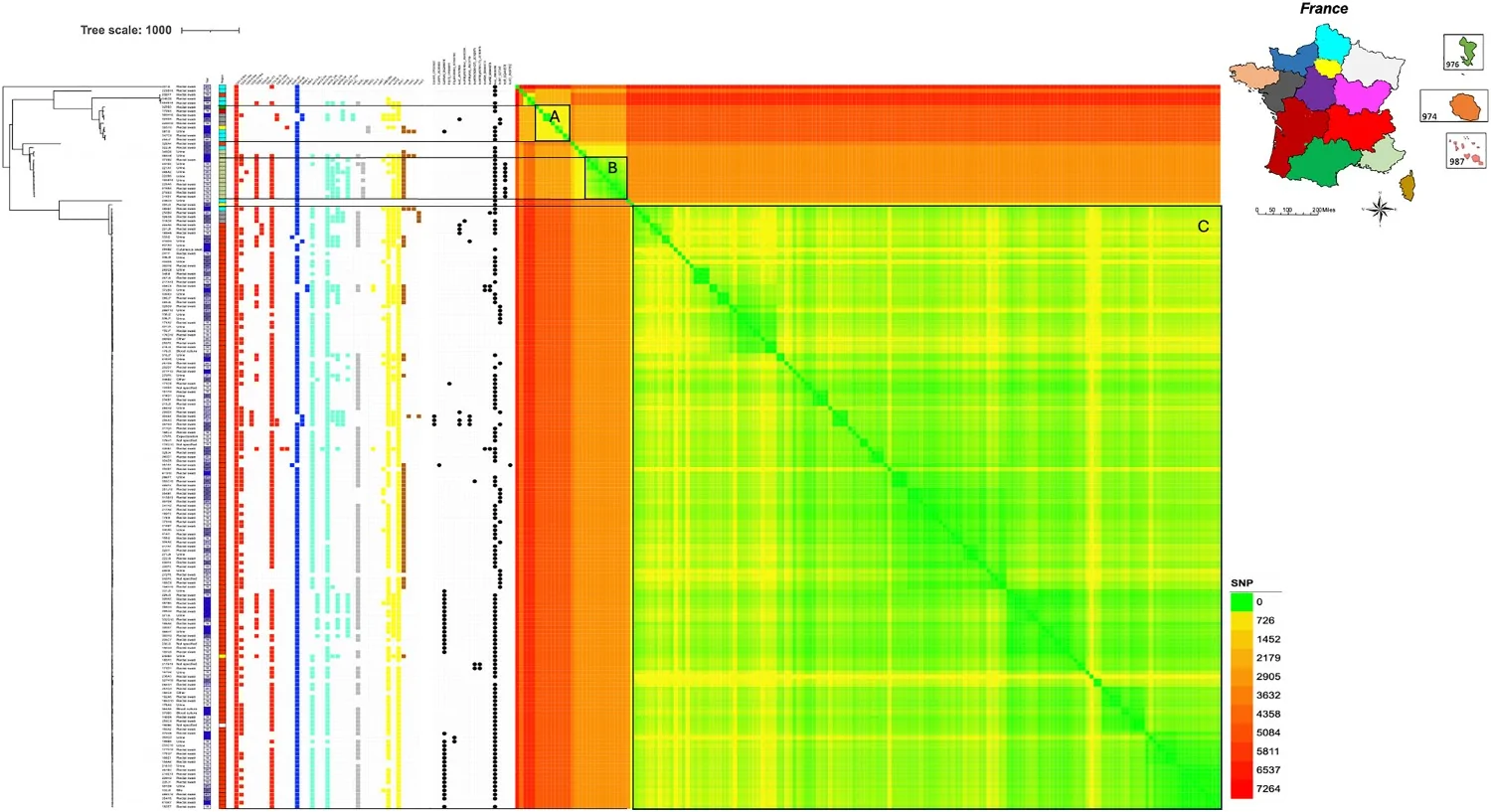
Phylogenic relationships and genomic features of *K. oxytoca* isolates belonging to ST-2. From left to right: phylogenetic tree, isolation site, year of collection, and geographical origin, followed by the presence/absence of antimicrobial resistance genes, virulence determinants, and plasmid replicase types. The right panel displays the pairwise SNP-distance matrix, where colour intensity reflects genetic relatedness between isolates (green = closely related, red = distant).

As *K. oxytoca* represented the vast majority of KoC isolates, subsequent analyses focused on this species to better characterize its population structure and genetic diversity. A total of 86 distinct sequence types (STs) were identified, with 38 had only a single representative isolate and 32 STs were represented by >2 isolated each (Supplementary Figure S3 Table S1). Five main sequence types (STs) were predominated including ST-2, ST-141, ST-19, ST-199, and ST-176 (Supplementary Figure S1 and S3). The two predominant lineages, ST-2 (n = 186) and ST-141 (n = 71), together accounting for nearly 40% of all KoC isolates (Figure S1 and S3). These two lineages were therefore selected for comparative analyses of their genetic background, resistance determinants, and phylogenetic relationships (Figure 2 and 3).

Within *K. oxytoca*, the ST-141 lineage comprised isolates recovered from diverse specimen types, including 39 rectal swabs, 22 urine samples, 3 blood cultures, 2 invasive specimen, 1 superficial sample, and 4 unspecified specimens (Figure 2). This sequence type was detected across multiple French regions between 2017 and 2023, highlighting its broad geographic dissemination. SNP-based phylogenetic analysis revealed a minimum of 0 and a maximum of 213 SNPs separating any two isolates within ST-141. Three main regional clusters were identified (Figure 2). Cluster α included four isolates from the Hauts-de-France region, showing fewer than 6 SNPs of difference. All produced NDM-1, CTX-M-15, AAC(3)-I, AAC(3)-II, ANT(3’)-IIa,APH(6)-Id, and Tet(A). Cluster β comprised seven isolates from the Auvergne–Rhône–Alpes region, with a maximum of 9 SNPs difference. All carried *bla*_OXA-48_, *aac(6’)-Ib*, and *tet(A)*; three also co-produced CTX-M-15, and five co-produced TEM-1. Cluster γ included 13 isolates, also from the Hauts-de-France region, with a maximum of 74 SNPs difference. All produced OXA-48, nine carried *aac(6’)-Ib*, and one carried *ant(3’)-IIa*. Within this ST-141 lineage, a substantial proportion of isolates also carried *merT* and *merC* genes, encoding membrane proteins involved in mercury transport (*merC*, *n* *=* *41*; *merT*, *n* *=* *40)*.

We next focused on the ST-2 lineage, which represented the dominant ST within the *K. oxytoca* population. A total of 186 isolates belonged to this lineage, of which 128 were recovered from rectal swabs, 43 from urine samples, 4 blood cultures, 2 from invasive specimen (including bile and bones), 2 respiratory samples, and 7 unspecified specimens (Figure 3). Within this lineage, the pairwise genomic distances ranged from 4 to 7,360 SNPs, including three major clusters within ST-2. In cluster A, 9 isolates showing a maximum of 268 SNPs of difference, including 7 OXA-48 produced and 2 NDM-1 plus CMY-2 producers. All originated from 5 distinct regions of France. Cluster B, comprised 10 isolates collected from Provence-Alpes-Cotes d’Azur regions and displayed a maximum of 31 SNPs of difference. All produce OXA-48, TEM-1 and CTX-M-15. The third and largest cluster (Cluster C) included 136 isolates with a maximum of 62 SNPs separating them. Within this cluster, 130 strains originated from the Auvergne-Rhône-Alpes region. 95,6% (n = 130) of isolates produced OXA-48, four produced NDM-1 (including one co-producing OXA-48), two produced OXA-181, and two produced VIM-4. Among the OXA-48-producing ST-2 isolates, 93.1% co-produced the extended-spectrum β-lactamase CTX-M-15. A more detailed analysis of cluster C identified three subclusters: C1 (19 isolates, ≤19 SNPs), C2 (16 isolates, ≤14 SNPs), and C3 (16 isolates, ≤16 SNPs), all corresponding to isolates recovered from the same hospital, suggesting ongoing patient-to-patient transmission within that facility (Supplementary Figure S4).

Given that ST-2 was by far the most prevalent lineage within the *K. oxytoca* population and was widely disseminated, particularly in the Auvergne–Rhône–Alpes region, we next sought to determine whether its epidemiological success could be linked to enhanced virulence or an increased ability to adhere to surfaces and form biofilm.

To investigate these hypotheses, we focused on *K. oxytoca* isolates obtained from long-term intestinal carriers, allowing the comparison of genetically related strains collected over time. Three patients exhibited prolonged colonization with OXA-48–producing *K. oxytoca*. Patient 1 was colonized for almost six years by ST-302 isolates (49C10, July 2014; 259A8, June 2020), which differed by only two SNPs. Patient 2 carried ST-2 isolates for 13 months, first detected in urine (133E9, March 2017) and later in a rectal screening sample (169J2, April 2018), differing by six SNPs. Patient 3 was colonized for seven months by ST-2 isolates (140D9, June 2017; 160A2, January 2018), which differed by four SNPs. Patients 2 and 3 were both hospitalized in the same hospital located near Lyon (Auvergne–Rhône–Alpes region, France). As controls, we included additional OXA-48–producing *K. oxytoca* isolates received at the F-NRC, belonging to various sequence types (ST-145, ST-30, ST-199, ST-2, ST-141, and ST-258) (Table S1).

Virulence-associated genes, including siderophore systems (enterobactin, yersiniabactin, salmochelin and aerobactin), fimbrial adhesins (*fim* and *mrk* gene clusters), and capsule-associated genes, were investigated and their prevalence compared between ST-2, ST-141 and the remaining sequence types. Overall, these virulence determinants were broadly distributed across the different lineages, with no major enrichment or depletion observed for most gene categories.

Interestingly, the salmochelin-associated gene *iroB* was highly prevalent among ST-141 isolates, being detected in 85.9% of strains, whereas it was absent from all ST-2 isolates and identified in only 10.8% of the remaining sequence types. Conversely, *hmoX*, a gene involved in heme-mediated iron acquisition, was rarely detected in ST-141 isolates (1.4%) but was highly prevalent in ST-2 isolates (92.9%) and showed an intermediate distribution among the other sequence types (45.4%) (Table S2).

### Virulence in *Galleria mellonella* model

**Figure 4.**
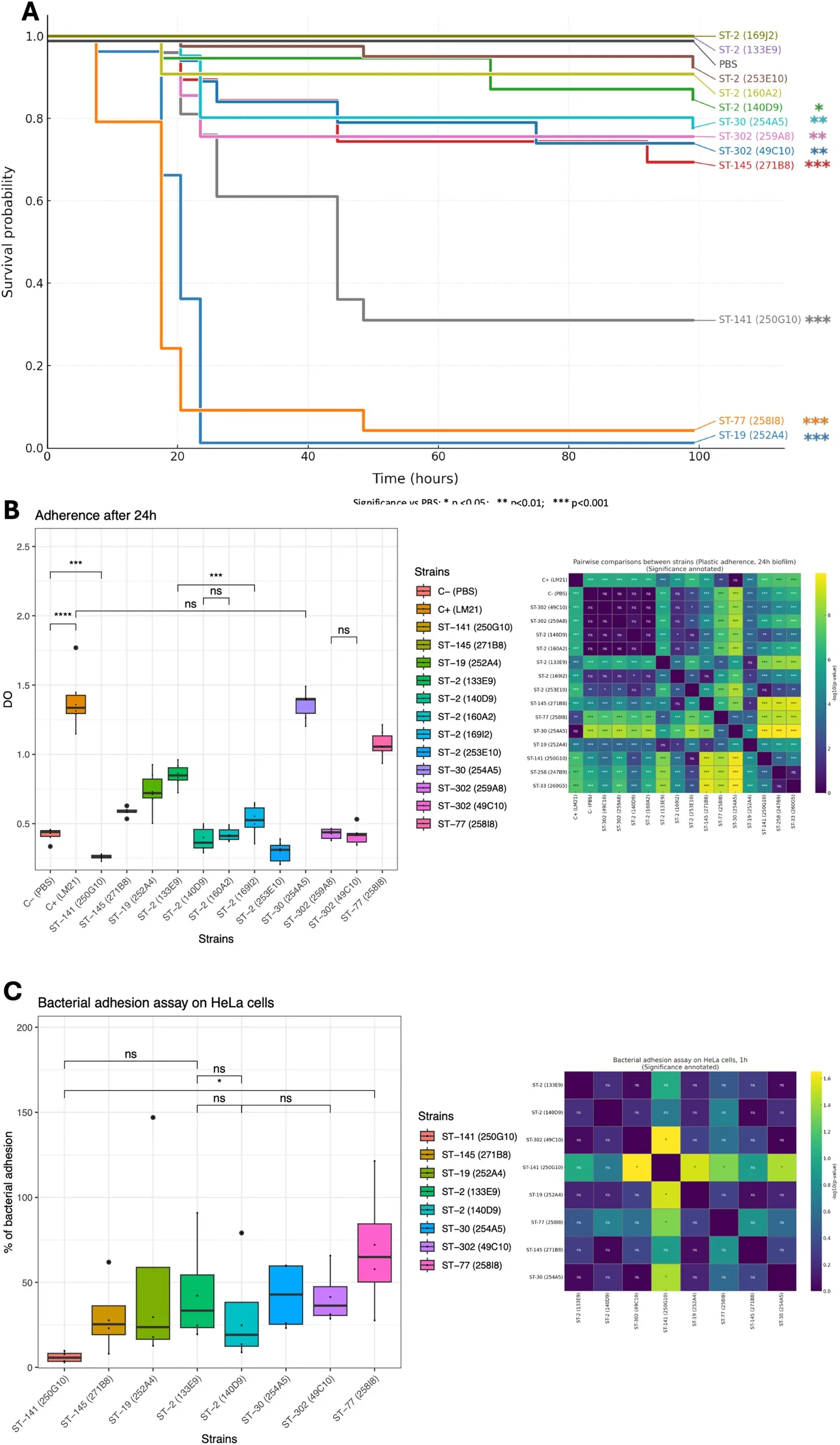
Virulence and adhesion/biofilm of OXA-48-producing *K. oxytoca* **A.** Virulence in *Galleria mellonela* model. *Galleria mellonella* (n = 10 per condition) were infected with clinical isolates belonging to ST-19 (252A4), ST-77 (258I8), ST-141 (250G10), ST-145 (271B8), ST-2 (133E9, 140D9, 160A2, 169J2, 252E10), and ST-302 (49C10, 259A8). PBS was used as negative control. Survival curves were monitored for 100 h. **B.** Adhesion/biofilm on polystyrene. Adherence was measured in 96-well plates using crystal violet staining, with optical density (OD) as readout after 24 of infection. Controls included PBS (C−) and the strong biofilm producer *K. pneumoniae* LM21 (C+). Boxplots show results for clinical isolates from different sequence types: ST-141 (250G10), ST-145 (271B8), ST-19 (252A4), ST-2 (133E9, 140D9, 160A2, 169J2, 252E10), ST-30 (254A5), ST-302 (49C10, 259A8), and ST-77 (258I8). Statistical analysis was performed by one-way ANOVA; ****p < 0.0001, *p < 0.05, ns = not significant. **C.** Adhesion on eukaryotic HeLa cells Adherence of OXA-48 producing *K. oxytoca* isolates to HeLa cells. Boxplots represent the percentage of bacterial adhesion relative to the inoculum for representative isolates belonging to ST-141 (250G10), ST-145 (271B8), ST-19 (252A4), ST-2 (133E9, 140D9), ST-30 (254A5), ST-302 (49C10), and ST-77 (258I8). Data are expressed as median with interquartile range, and dots indicate individual replicates. Statistical comparisons were performed using one-way ANOVA with multiple comparisons; ns = not significant.

To assess virulence, *Galleria mellonella* larvae were infected with clinical isolates. PBS was used as a negative control, confirming that larvae remained alive throughout the experiment (Figure 4A). As expected, isolates collected longitudinally from the same patient displayed comparable virulence profiles (169J2 and 133E9; 160A2 and 140D9; 259A8 and 49C10), supporting the reproducibility of our assay. A striking finding was the absence of virulence among ST-2 isolates, whose survival curves did not differ significantly from those of the PBS control, including for the non-clonal isolate 252E10. In contrast, ST-19 (252A4) and ST-77 (258I8) exhibited the highest virulence, killing 50% of larvae within 21 h, with a significant difference compared to PBS. ST-141 (250G10) also demonstrated strong virulence, reaching 50% mortality within 40 h.

### Biofilm formation and adherence on abiotic surfaces

The ability of isolates to adhere and form biofilm was assessed in 96-well plates (Figure 4B). PBS and the K. pneumoniae LM21 strain (known as high biofilm producer) served as negative and positive controls, respectively, and differed markedly (*p* < 0.0001). Among clinical isolates, ST-30 (254A5) produced biofilm at a level comparable to the positive control (no significative difference, ns), followed by ST-77 (258I8). In contrast, ST-141 (250G10) was the least adherent strain. For ST-2 isolates from long-term carriers, no significant differences were detected for patients 1 and 3 (ns), whereas a significant difference was observed for patient 2 between the two time points (*p* < 0.001), with the earlier isolate producing more biofilm. Overall, the ST-2 and ST-302 isolates that colonized patients over prolonged periods did not behave as strong biofilm formers on abiotic surfaces – unlike ST-30, which adhered as much as the positive control.

### Adherence to HeLa cells

Adhesion to epithelial cells was further evaluated using HeLa cells (Figure 4C). ST-2/ST-302 isolates from patient 1 (49C10), patient 2 (133E9) and patient 3 (140D9) showed no significant differences. ST-141 (250G10) exhibited very limited adhesion, whereas ST-77 (258I8) displayed the strongest adherence phenotype (*p* value<0,05).

## Discussion

To our knowledge, this study represents the largest investigation to date focusing on carbapenemase-producing *Klebsiella oxytoca* complex (KoC) isolates. All isolates were collected nationwide through the French-NRC, providing a comprehensive overview of the epidemiology, genetic diversity, and virulence potential of KoC across France. Among the six closely related species of the KoC*, K. oxytoca* was by far the most prevalent in our collection, followed by *K. michiganensis* and *K. grimontii*, in agreement with previous genomic surveys [11,27].

The distribution of carbapenemases across the different KoC species is particularly noteworthy for *K. michiganensis* (and *K. michiganensis*-like isolates), which preferentially harbour VIM enzymes. This has already been highlighted in a recent Taiwanese study [28]. However, *K. oxytoca* remains the most represented species in our collection and is overwhelmingly dominated by OXA-48–like carbapenemases, mirroring both national and European epidemiology. Despite the high genetic heterogeneity observed across the complex, *K. oxytoca* displayed a clear clonal structure dominated by two closely related lineages, ST-141 and ST-2. The ST-141 lineage has not been reported in previous studies; its predominance in our dataset likely reflects regional dissemination in the Hauts-de-France and Auvergne–Rhône–Alpes areas, forming a localized cluster. Conversely, ST-2 has been described as a prevalent clinical lineage previously associated with the dissemination of carbapenemases [29–31], although the enzyme types differed from those reported here [30]. Notably, ST-2 isolates were mainly recovered from the Auvergne–Rhône–Alpes region, raising questions about the factors contributing to the success and persistence of this clone, as previously observed in long-term colonization cases [31].

To explore these traits, we analysed the virulence and adhesion profiles of OXA-48–producing *K. oxytoca* isolates. ST-2 showed the lowest virulence and limited biofilm formation, consistent with its ability to persist in patients over time. This phenotype may promote silent colonization rather than overt infection, favouring long-term dissemination. Similar findings have been reported for ST-2 *KoC* strains producing KPC-2, −3, and IMP-8 in hospital environments [31], as well as for OXA-48–producing *K. oxytoca* in Tunisia, where transmission occurred without ESBL co-expression, highlighting diagnostic and surveillance challenges [4].

In contrast, ST-141 displayed an intermediate phenotype, characterized by moderate virulence and no detectable adhesion or biofilm formation. Interestingly, ST-141 isolates harboured *merB*, *merC*, and *merT* genes encoding mercury reductase enzymes [32], which might confer an ecological advantage by enhancing survival in contaminated environments [33]. In addition, the two dominant lineages displayed distinct iron acquisition profiles. ST-141 was strongly associated with iroB, a key component of the salmochelin system and a recognized marker of hypervirulent *Klebsiella pneumoniae* lineages [34], whereas this gene was absent from ST-2 isolates. Conversely, ST-2 was enriched in hmoX, which encodes a heme oxygenase enabling the utilization of host-derived heme as an iron source [35]. These differences are consistent with the phenotypic characteristics observed in our study, where ST-2 combined low virulence with high prevalence and prolonged colonization. Enhanced heme utilization may therefore represent one of the mechanisms contributing to the persistence and ecological success of this lineage, while ST-141 appears to rely on a distinct iron acquisition strategy more commonly associated with virulence. Although ST-141 has not been described as a high-risk clone, several small clusters were identified in the Hauts-de-France and Auvergne–Rhône–Alpes regions, suggesting regional dissemination rather than large-scale spread. Its apparent success in our collection may therefore reflect local ecological adaptation and transient expansion, rather than the emergence of a virulent or epidemic lineage.

By contrast, ST-19 and ST-77, which are not predominant STs, exhibited high virulence in the *Galleria mellonela* infection model, causing rapid mortality in larvae. Such marked pathogenicity may reduce their ability to persist or disseminate, as excessive virulence can hinder long-term colonization.

Altogether, these findings indicate that the epidemiological success of *K. oxytoca* complex lineages is not solely driven by antibiotic resistance but also shaped by the interplay between virulence and colonization dynamics. Although French surveillance efforts mainly target *K. pneumoniae* carbapenemases producers, our results emphasize the importance of extending genomic monitoring to carbapenemase–producing *K. oxytoca.* The coexistence of persistent, low-virulence lineages such as ST-2 and more virulent or environmentally adapted ones like ST-141 highlights the diverse evolutionary strategies contributing to the dissemination of carbapenemase-producing *K. oxytoca* in France.

From a genomic perspective, future work should focus on a more detailed characterization of iron acquisition systems, particularly the salmochelin-associated iro locus and the heme utilization gene hmoX, which displayed contrasting distributions between the two dominant lineages. Investigating the genetic organization, regulation, and functional impact of these determinants may provide further insight into the mechanisms underlying the distinct virulence and persistence phenotypes observed among *K. oxytoca* lineages.

Disclosure statementNo potential conflict of interest was reported by the author(s).

## Supplementary Material

Table S2_revised.pdf

Figure S4.pdf

Figure S2.pdf

Table S1_revised.pdf

Figure S3.pdf

Figure S1.pdf
