# Supplementary material for: Carbapenemase-producing Klebsiella oxytoca complex in France (2017–2023): molecular epidemiology integrated with virulence and biofilm phenotypes
Source: Emerg Microbes Infect. 2026 Aug 3;15(1):2713319. doi: 10.1080/22221751.2026.2713319 (PMC13508490; doi:10.1080/22221751.2026.2713319)
Supplement: Supplementary file 3 [file TEMI_A_2713319_SM6353.pdf]

Strain	Species	ST - Oxford	Carbapenemase	ESBL	Acquired cephalosporinase	year of isolation	Clinical sample	Strains used for adhesion / biofilm assays	Strains used in *Galleria mellonela* model
113C1	Klebsiella oxytoca	34	OXA-48	/	/	2016	Urine	No	No
113J6	Klebsiella oxytoca	36	VIM-1	/	/	2016	Rectal Swab	No	No
124F2	Klebsiella grimontii	168	OXA-48	/	/	2016	Rectal Swab	No	No
130F4	Klebsiella oxytoca	223	OXA-48	/	/	2017	Rectal Swab	No	No
129D10	Klebsiella oxytoca	223	OXA-48	/	/	2017	Urine	No	No
151D10	Klebsiella pasteurii	185	KPC-2	/	CMY-4	2017	Rectal Swab	No	No
155E3	Klebsiella michiganensis	211	KPC-2	/	/	2017	Urine	No	No
159H3	Klebsiella oxytoca	199	OXA-48	/	/	2018	not determined	No	No
161B3	Klebsiella michiganensis	135	NDM-1	SHV-12	CMY-4	2018	Rectal Swab	No	No
164C2	Klebsiella oxytoca	152	OXA-48	CTX-M-15	/	2018	Rectal Swab	No	No
167I8	Klebsiella oxytoca	141	OXA-48	/	/	2018	Wound	No	No
169G3	Klebsiella michiganensis	211	KPC-2	/	/	2018	Rectal Swab	No	No
169J2	Klebsiella oxytoca	2	OXA-48	/	/	2018	Rectal Swab	Yes	Yes
170E6	Klebsiella michiganensis	180	OXA-48	/	/	2018	Rectal Swab	No	No
174C9	Klebsiella oxytoca	746	OXA-48	/	/	2018	Wound	No	No
174I3	Klebsiella oxytoca	345	OXA-48	/	/	2018	Rectal Swab	No	No
176G9	Klebsiella oxytoca	759	OXA-48	/	/	2018	Rectal Swab	No	No
176H7	Klebsiella oxytoca	176	VIM-1	SHV-12	/	2018	not determined	No	No
177A1	Klebsiella oxytoca	496	OXA-48	/	/	2018	Rectal Swab	No	No
181I5	Klebsiella oxytoca	2-like	OXA-48	CTX-M-15	/	2018	Rectal Swab	No	No
185G10	Klebsiella oxytoca	2	OXA-48	CTX-M-15	/	2018	Rectal Swab	No	No
191A7	Klebsiella oxytoca	59	OXA-48	/	/	2018	Rectal Swab	No	No
196A6	Klebsiella oxytoca	19	OXA-48	CTX-M-15	/	2018	Rectal Swab	No	No
202D7	Klebsiella oxytoca	2	OXA-48	CTX-M-15	/	2019	Rectal Swab	No	No
204F2	Klebsiella oxytoca	19	OXA-48	CTX-M-15	/	2019	Urine	No	No
204I4	Klebsiella oxytoca	19	OXA-48	CTX-M-15	/	2019	Urine	No	No
212H3	Klebsiella oxytoca	141	OXA-48	/	/	2019	Urine	No	No
213C3	Klebsiella michiganensis	35	OXA-48	/	/	2019	Rectal Swab	No	No
213F7	Klebsiella grimontii	618	OXA-48	/	/	2019	Rectal Swab	No	No
215E1	Klebsiella oxytoca	2	OXA-48	CTX-M-15	/	2019	Rectal Swab	No	No
215E2	Klebsiella oxytoca	2	OXA-48	CTX-M-15	/	2019	Rectal Swab	No	No
217C4	Klebsiella grimontii	861-like	OXA-48	/	/	2019	Rectal Swab	No	No
217E10	Klebsiella oxytoca	2	OXA-48	/	/	2019	Rectal Swab	No	No
218A10	Klebsiella oxytoca	288	OXA-48	/	/	2019	Rectal Swab	No	No
220I4	Klebsiella oxytoca	179-like	OXA-48	/	/	2019	Rectal Swab	No	No
221D5	Klebsiella oxytoca	111-like	OXA-48	CTX-M-15	/	2019	Rectal Swab	No	No
222E6	Klebsiella oxytoca	285	OXA-48	/	/	2019	Rectal Swab	No	No
222J9	Klebsiella oxytoca	2	OXA-48	CTX-M-15	/	2019	Rectal Swab	No	No
223F8	Klebsiella oxytoca	375-like	OXA-48	/	/	2019	Rectal Swab	No	No
225C6	Klebsiella michiganensis	35-like	NDM-5	/	/	2019	Vaginal Swab	No	No
225J3	Klebsiella oxytoca	59	OXA-48	/	/	2019	Rectal Swab	No	No
226C2	Klebsiella oxytoca	258	OXA-181	/	/	2019	Rectal Swab	No	No
227A3	Klebsiella oxytoca	58	OXA-48	/	/	2019	Rectal Swab	No	No
227B4	Klebsiella grimontii	168	OXA-48	SHV-12	/	2019	Rectal Swab	No	No
228G5	Klebsiella grimontii	404-like	OXA-48	/	DHA-1	2019	Rectal Swab	No	No
230C9	Klebsiella oxytoca	111-like	OXA-48	/	/	2019	Rectal Swab	No	No
231A2	Klebsiella oxytoca	58	OXA-48	CTX-M-15	/	2019	Rectal Swab	No	No
231F4	Klebsiella grimontii	215	OXA-48	CTX-M-15	/	2019	Rectal Swab	No	No
233A7	Klebsiella oxytoca	179-like	OXA-48	/	/	2019	Urine	No	No
233C9	Klebsiella oxytoca	19	OXA-48	/	/	2019	Rectal Swab	No	No
233F2	Klebsiella grimontii	172	VIM-1	/	ACC-1, LAP-2	2019	Rectal Swab	No	No
236G8	Klebsiella michiganensis	135	OXA-48	/	/	2019	Rectal Swab	No	No
236H3	Klebsiella oxytoca	58	OXA-48	/	/	2019	Rectal Swab	No	No
236H6	Klebsiella oxytoca	19	OXA-48	CTX-M-15	/	2019	Urine	No	No
241E2	Klebsiella michiganensis	88	OXA-48	/	/	2019	Rectal Swab	No	No
241H2	Klebsiella oxytoca	2	OXA-48	CTX-M-15	/	2019	Rectal Swab	No	No
241I1	Klebsiella oxytoca	2	Non CPE	CTX-M-15	/	2019	Rectal Swab	No	No
247B9	Klebsiella oxytoca	258	OXA-48	/	/	2020	Urine	No	No
247E1	Klebsiella oxytoca	19	OXA-48	/	/	2020	Urine	No	No
248A3	Klebsiella michiganensis	135	OXA-48	CTX-M-15	/	2020	not determined	No	No
252A4	Klebsiella oxytoca	19	OXA-48	CTX-M-15	/	2020	Blood culture	Yes	Yes
254A5	Klebsiella oxytoca	30	OXA-48	/	/	2020	Rectal Swab	Yes	Yes
254J10	Klebsiella oxytoca	287	NDM-1	CTX-M-15	/	2020	Rectal Swab	No	No
256G9	Klebsiella oxytoca	19	OXA-48	CTX-M-15	/	2020	Urine	No	No
257G6	Klebsiella grimontii	184	OXA-48	/	/	2020	Rectal Swab	No	No
259F3	Klebsiella oxytoca	288	OXA-48	/	/	2020	Urine	No	No
260G5	Klebsiella oxytoca	33	OXA-48	/	/	2020	Rectal Swab	No	No
263G7	Klebsiella oxytoca	237	OXA-48	CTX-M-15	/	2020	Rectal Swab	No	No
265F5	Klebsiella grimontii	362	OXA-48	/	/	2020	Rectal Swab	No	No
268D6	Klebsiella grimontii	172	VIM-1	/	ACC-1, LAP-2	2020	Urine	No	No
267E9	Klebsiella oxytoca	19	OXA-48	CTX-M-15	/	2020	Urine	No	No
270B7	Klebsiella oxytoca	145	OXA-48	CTX-M-15	/	2020	Urine	No	No
271B8	Klebsiella oxytoca	145	OXA-48	CTX-M-15	/	2020	Urine	Yes	Yes
271J9	Klebsiella oxytoca	2	OXA-48	CTX-M-15	/	2020	Urine	No	No
272F8	Klebsiella oxytoca	2	OXA-48	/	/	2020	Urine	No	No
272G1	Klebsiella oxytoca	141	OXA-48	/	/	2020	Rectal Swab	No	No
274C7	Klebsiella michiganensis	88	OXA-48	CTX-M-15	/	2020	Urine	No	No
276G7	Klebsiella michiganensis	135	OXA-48	/	/	2021	Rectal Swab	No	No
160A2	Klebsiella oxytoca	2	OXA-48	CTX-M-15	/	2018	Rectal Swab	Yes	Yes
277H7	Klebsiella michiganensis	135	OXA-48	/	/	2021	Rectal Swab	No	No
277I8	Klebsiella oxytoca	19	OXA-48	CTX-M-15	/	2021	Urine	No	No
278C3	Klebsiella oxytoca	364	OXA-48	/	/	2021	Rectal Swab	No	No
278H5	Klebsiella oxytoca	178	OXA-48	CTX-M-9	/	2021	Urine	No	No
280F7	Klebsiella grimontii	263	OXA-48	/	/	2021	Rectal Swab	No	No

281G3	Klebsiella michiganensis	135	OXA-48	CTX-M-15	/	2021	Rectal Swab	No	No
282B7	Klebsiella oxytoca	145	OXA-48	CTX-M-15	/	2021	Rectal Swab	No	No
282D5	Klebsiella oxytoca	111-like	OXA-48	/	/	2021	Rectal Swab	No	No
286B7	Klebsiella oxytoca	120-like	OXA-48	/	/	2021	Urine	No	No
286F10	Klebsiella oxytoca	2	OXA-48	/	/	2021	Urine	No	No
287A5	Klebsiella oxytoca	302-like	OXA-48	/	/	2021	Urine	No	No
34F1	Klebsiella oxytoca	152	NDM-1	CTX-M-15, SHV-12	/	2013	Other	No	No
287H3	Klebsiella oxytoca	19	OXA-48	CTX-M-15	/	2021	Respiratory sample	No	No
287J7	Klebsiella oxytoca	601-like	OXA-48	/	/	2021	Rectal Swab	No	No
259A8BIS	Klebsiella oxytoca	302	OXA-48	/	/	2020	Rectal Swab	Yes	Yes
288C2	Klebsiella grimontii	618	NDM-1	SHV-12	CMY-4	2021	Rectal Swab	No	No
290I9	Klebsiella oxytoca	2	OXA-48	/	/	2021	Urine	No	No
291I5	Klebsiella oxytoca	3-like	OXA-48	/	/	2021	Urine	No	No
292G9	Klebsiella oxytoca	141	OXA-48	CTX-M-15	/	2021	Urine	No	No
292H1	Klebsiella grimontii	71-like	NDM-1	SHV-12	CMY-4	2021	Rectal Swab	No	No
294G10	Klebsiella oxytoca	141	OXA-48	CTX-M-15	/	2021	Urine	No	No
295I8	Klebsiella oxytoca	237	OXA-48	CTX-M-15	/	2021	Rectal Swab	No	No
296F7	Klebsiella oxytoca	2	OXA-48	CTX-M-15	/	2021	Urine	No	No
296I7	Klebsiella oxytoca	141-like	OXA-48	/	/	2021	Rectal Swab	No	No
296J2	Klebsiella oxytoca	145	OXA-48	CTX-M-15	/	2021	Rectal Swab	No	No
297D6	Klebsiella oxytoca	145	OXA-48	CTX-M-15	/	2021	Urine	No	No
298J2	Klebsiella oxytoca	19	OXA-48	/	/	2021	Urine	No	No
299F2	Klebsiella oxytoca	2	OXA-48	CTX-M-15	/	2021	Rectal Swab	No	No
301B2	Klebsiella oxytoca	30	OXA-48	/	/	2021	Rectal Swab	No	No
300A5BIS	Klebsiella grimontii	168	OXA-48	CTX-M-15	/	2021	Respiratory sample	No	No
302A7	Klebsiella oxytoca	58	OXA-48	/	/	2021	Rectal Swab	No	No
302I6	Klebsiella oxytoca	257	OXA-48	/	/	2021	Urine	No	No
304B10	Klebsiella oxytoca	141	OXA-48	/	/	2021	Blood culture	No	No
304C2	Klebsiella michiganensis	35-like	OXA-48	CTX-M-15	/	2021	Urine	No	No
304D5	Klebsiella oxytoca	2	OXA-48	/	/	2021	Rectal Swab	No	No
304E4	Klebsiella oxytoca	2	NDM-1	CTX-M-15	/	2021	Rectal Swab	No	No
307J7	Klebsiella michiganensis	38-like	OXA-48	/	/	2021	Urine	No	No
310J9	Klebsiella oxytoca	179	OXA-48	/	/	2022	Urine	No	No
314I3	Klebsiella oxytoca	141	Non CPE	CTX-M-15	/	2022	Urine	No	No
315H2	Klebsiella grimontii	291	OXA-48	/	/	2022	Rectal Swab	No	No
317B10	Klebsiella grimontii	490	OXA-48	CTX-M-15	/	2022	Rectal Swab	No	No
317H8	Klebsiella michiganensis	138-like	NDM-1	CTX-M-15	/	2022	Rectal Swab	No	No
318B1	Klebsiella oxytoca	237	OXA-48	CTX-M-15	/	2022	Respiratory sample	No	No
318H6	Klebsiella oxytoca	176-like	Non CPE	CTX-M-15	/	2022	Urine	No	No
320G10	Klebsiella michiganensis	180	NDM-5	/	/	2022	Rectal Swab	No	No
321J10	Klebsiella grimontii	291	OXA-48	/	/	2022	Rectal Swab	No	No
322D8	Klebsiella grimontii	467	OXA-48	/	/	2022	Urine	No	No
324J8	Klebsiella oxytoca	19-like	OXA-48	/	/	2022	Urine	No	No
326A4	Klebsiella oxytoca	2	OXA-48	/	/	2022	Rectal Swab	No	No
326D6	Klebsiella oxytoca	19	OXA-48	CTX-M-15	/	2022	Rectal Swab	No	No
330F2	Klebsiella michiganensis	88	OXA-48	CTX-M-3	/	2022	Rectal Swab	No	No
330I8	Klebsiella oxytoca	288	OXA-48	/	/	2022	Rectal Swab	No	No
330J2	Klebsiella grimontii	263	NDM-1	CTX-M-15	/	2022	Rectal Swab	No	No
331A7	Klebsiella grimontii	214-like	VIM-4	/	/	2022	Urine	No	No
328B7BIS	Klebsiella oxytoca	237	OXA-48	CTX-M-15	/	2022	Urine	No	No
331D1	Klebsiella oxytoca	237	OXA-48	CTX-M-15	/	2022	Urine	No	No
333I1	Klebsiella oxytoca	176	NDM-1	SHV-12	CMY-4	2022	Wound	No	No
335K6	Klebsiella grimontii	327	OXA-48	CTX-M-15	/	2022	Rectal Swab	No	No
343C10	Klebsiella oxytoca	19-like	OXA-48	/	/	2022	Rectal Swab	No	No
345F2	Klebsiella michiganensis	135	OXA-48	CTX-M-15	/	2022	Rectal Swab	No	No
348J8	Klebsiella oxytoca	141	OXA-48	/	/	2022	Rectal Swab	No	No
354D3	Klebsiella oxytoca	258-like	NDM-1	SHV-12	CMY-4	2022	Rectal Swab	No	No
354G4	Klebsiella oxytoca	289	OXA-48	/	/	2022	Rectal Swab	No	No
355B10	Klebsiella oxytoca	258-like	NDM-1	SHV-12	CMY-4	2022	Respiratory sample	No	No
357A1	Klebsiella oxytoca	2	OXA-181	CTX-M-15	/	2022	Rectal Swab	No	No
358B10	Klebsiella oxytoca	58	OXA-48	CTX-M-15	/	2022	Rectal Swab	No	No
358J2	Klebsiella oxytoca	199	OXA-48	/	/	2022	Rectal Swab	No	No
359J2	Klebsiella oxytoca	58	OXA-48	CTX-M-15	/	2022	Respiratory sample	No	No
362I2	Klebsiella oxytoca	48-like	OXA-48	/	/	2023	Urine	No	No
367C9	Klebsiella oxytoca	58	VIM-1	/	ACC-1	2023	Rectal Swab	No	No
373J7	Klebsiella grimontii	361-like	OXA-48	/	/	2023	Rectal Swab	No	No
375K3	Klebsiella oxytoca	223	NDM-1	CTX-M-15	/	2023	Undetermined	No	No
376I1	Klebsiella oxytoca	237	OXA-48	/	/	2023	Urine	No	No
376J6	Klebsiella oxytoca	19	OXA-48	/	/	2023	Rectal Swab	No	No
377B8	Klebsiella oxytoca	19	OXA-48	CTX-M-15	/	2023	Urine	No	No
378A1	Klebsiella oxytoca	258	NDM-1	/	/	2023	Rectal Swab	No	No
381K9	Klebsiella oxytoca	223	OXA-48	/	/	2023	Rectal Swab	No	No
382A3	Klebsiella pasteurii	386	NDM-1	SHV-12	CMY-4	2023	Rectal Swab	No	No
382C6	Klebsiella oxytoca	199	OXA-48	CTX-M-14	/	2023	Rectal Swab	No	No
384B1	Klebsiella grimontii	800	OXA-48	/	/	2023	Rectal Swab	No	No
385G1	Klebsiella grimontii	129-like	VIM-1 + OXA-48	SHV-12	/	2023	Rectal Swab	No	No
385J4	Klebsiella oxytoca	19-like	OXA-48	/	/	2023	Urine	No	No
386F1	Klebsiella grimontii	327	NDM-1	CTX-M-15	/	2023	Rectal Swab	No	No
387A1	Klebsiella michiganensis	135	VIM-1	/	/	2023	Rectal Swab	No	No
387J10	Klebsiella oxytoca	145-like	NDM-1	CTX-M-15	/	2023	Urine	No	No
389B4	Klebsiella oxytoca	58	OXA-48	CTX-M-15	/	2023	Rectal Swab	No	No
392I6	Klebsiella oxytoca	258	NDM-1	/	/	2023	Rectal Swab	No	No
395B4	Klebsiella grimontii	129-like	VIM-1 + OXA-48	SHV-12	/	2023	Rectal Swab	No	No
399J1	Klebsiella grimontii	618	VIM-1	SHV-12	/	2023	Rectal Swab	No	No
400A9	Klebsiella oxytoca	48	OXA-48	/	/	2023	Rectal Swab	No	No
404D3	Klebsiella oxytoca	19	NDM-5 + OXA-48	CTX-M-15	/	2023	Rectal Swab	No	No
404F1	Klebsiella grimontii	129-like	OXA-48	/	/	2023	Urine	No	No
411D6	Klebsiella oxytoca	237	OXA-48	CTX-M-15	/	2023	Urine	No	No

416F7	Klebsiella oxytoca	199	NDM-1	CTX-M-15	/	2023	Rectal Swab	No	No
420B1	Klebsiella oxytoca	19-like	OXA-48	CTX-M-15	/	2023	Rectal Swab	No	No
420B2	Klebsiella oxytoca	375	OXA-48	/	/	2023	Rectal Swab	No	No
146A4	Klebsiella michiganensis-like	82	KPC-2	/	/	2017	Rectal Swab	No	No
149J7	Klebsiella michiganensis	43	OXA-181	/	/	2017	Undetermined	No	No
149J8	Klebsiella michiganensis	108-like	OXA-48	/	/	2017	Undetermined	No	No
161I8	Klebsiella grimontii	408	OXA-48	/	/	2018	Rectal Swab	No	No
163F1	Klebsiella oxytoca	141	NDM-1	CTX-M-15	/	2018	Rectal Swab	No	No
166G9	Klebsiella michiganensis-like	310	VIM-1	CTX-M-36	/	2018	Undetermined	No	No
167E9	Klebsiella michiganensis	98	OXA-48	/	/	2018	Rectal Swab	No	No
173H9	Klebsiella oxytoca	2	OXA-48	CTX-M-15	/	2018	Rectal Swab	No	No
175E4	Klebsiella oxytoca	58	OXA-48	/	/	2018	Rectal Swab	No	No
177D7	Klebsiella michiganensis	354	NDM-1	SHV-12	CMY-4	2018	Rectal Swab	No	No
184C9	Klebsiella michiganensis	202	VIM-4	/	CMY-4	2018	Rectal Swab	No	No
185C9	Klebsiella oxytoca	2	OXA-48	CTX-M-15	/	2018	Other	No	No
188J8	Klebsiella michiganensis-like	197	OXA-48	/	/	2018	Rectal Swab	No	No
191A1	Klebsiella michiganensis-like	95	OXA-48	/	/	2018	Rectal Swab	No	No
206E3	Klebsiella michiganensis	108	Non CPE	CTX-M-15	/	2019	Rectal Swab	No	No
207E7	Klebsiella michiganensis	511-like	NDM-1	/	/	2019	Rectal Swab	No	No
209D2	Klebsiella michiganensis	146	OXA-48	CTX-M-15, SHV-12	/	2019	Rectal Swab	No	No
210H9	Klebsiella michiganensis	433	VIM-1	/	ACC-1	2019	Rectal Swab	No	No
211D8	Klebsiella oxytoca	141-like	OXA-48	/	/	2019	Rectal Swab	No	No
215E3	Klebsiella oxytoca	2	OXA-48	CTX-M-15	/	2019	Rectal Swab	No	No
215J4	Klebsiella michiganensis	860-like	OXA-181	/	/	2019	Urine	No	No
222B8	Klebsiella oxytoca	2	OXA-48	CTX-M-15	/	2019	Urine	No	No
225B3	Klebsiella michiganensis	354	OXA-48	/	CMY-4	2019	Rectal Swab	No	No
225F8	Klebsiella oxytoca	18	OXA-48	CTX-M-15	/	2019	Urine	No	No
226C1	Klebsiella michiganensis	52	OXA-48	/	/	2019	Rectal Swab	No	No
228H9	Klebsiella huaxiensis	875	OXA-48	/	/	2019	Rectal Swab	No	No
229G2	Klebsiella michiganensis	205	OXA-48	/	/	2019	Rectal Swab	No	No
233E1	Klebsiella michiganensis	435	OXA-48	CTX-M-15	/	2019	Wound	No	No
234C3	Klebsiella michiganensis	11	VIM-1	/	/	2019	Respiratory sample	No	No
235A7	Klebsiella michiganensis	52	OXA-48	/	/	2019	Rectal Swab	No	No
235C1	Klebsiella oxytoca	36	OXA-48	/	/	2019	Rectal Swab	No	No
235I2	Klebsiella spallanzanii	876-like	VIM-1	/	/	2019	Rectal Swab	No	No
237F5	Klebsiella oxytoca	111-like	OXA-48	/	/	2019	Rectal Swab	No	No
237F6	Klebsiella michiganensis-like	310	OXA-48	CTX-M-15	/	2019	Rectal Swab	No	No
250A1	Klebsiella michiganensis	609-like	OXA-48	/	/	2020	Rectal Swab	No	No
251J7	Klebsiella oxytoca	848-like	OXA-48	/	/	2020	Other	No	No
253D3	Klebsiella oxytoca	364	NDM-7	/	/	2020	Urine	No	No
253E2	Klebsiella michiganensis	52	OXA-48	/	/	2020	Other	No	No
255B1	Klebsiella pasteurii	608	OXA-48	CTX-M-15	/	2020	Urine	No	No
256C8	Klebsiella michiganensis-like	310	VIM-1	/	/	2020	Rectal Swab	No	No
257D1	Klebsiella oxytoca	364	OXA-48	/	/	2020	Urine	No	No
258I8	Klebsiella michiganensis-like	77	OXA-48	/	/	2020	Rectal Swab	Yes	Yes
259J7	Klebsiella oxytoca	2	OXA-48	/	/	2020	Rectal Swab	No	No
260D1	Klebsiella oxytoca	2	OXA-48	CTX-M-15	/	2020	Rectal Swab	No	No
261B7	Klebsiella michiganensis-like	722	NDM-4 + VIM-1	/	/	2020	Rectal Swab	No	No
261G5	Klebsiella michiganensis	52	VIM-1	/	/	2020	Urine	No	No
264C8	Klebsiella michiganensis-like	29	VIM-1	/	/	2020	Blood culture	No	No
275A6	Klebsiella michiganensis	27-like	NDM-1	CTX-M-15	/	2020	Rectal Swab	No	No
275I7	Klebsiella michiganensis-like	427	NDM-1	CTX-M-15	/	2020	Rectal Swab	No	No
279C1	Klebsiella oxytoca	176,0	OXA-48	/	/	2021	Rectal Swab	No	No
279E7BIS	Klebsiella oxytoca	223	OXA-48	SHV-12, CTX-M-9	/	2021	Rectal Swab	No	No
281A10	Klebsiella michiganensis	324	VIM-4	/	/	2021	Other	No	No
283E7	Klebsiella oxytoca	201-like	OXA-48	/	/	2021	Respiratory sample	No	No
286B8	Klebsiella oxytoca	201	OXA-48	/	/	2021	Urine	No	No
286H10	Klebsiella michiganensis-like	310	VIM-1	/	/	2021	Rectal Swab	No	No
287B9	Klebsiella michiganensis	144	NDM-1	CTX-M-15	ACC-1	2021	Respiratory sample	No	No
243J9	Klebsiella oxytoca	141-like	NDM-1	/	/	2020	Rectal Swab	No	No
287H5	Klebsiella oxytoca	141	OXA-48	/	/	2021	Urine	No	No
49C10BIS	Klebsiella oxytoca	302	OXA-48	/	/	2013	Rectal Swab	Yes	Yes
288C10	Klebsiella oxytoca	2	OXA-48	CTX-M-15	/	2021	Rectal Swab	No	No
288I10	Klebsiella michiganensis	324	VIM-4	/	/	2021	Other	No	No
289C10	Klebsiella michiganensis	135	OXA-48	CTX-M-15	/	2021	Rectal Swab	No	No
289G5	Klebsiella oxytoca	141	OXA-48	/	/	2021	Urine	No	No
289J6	Klebsiella oxytoca	2	OXA-48	CTX-M-15	/	2021	Rectal Swab	No	No
292I4	Klebsiella michiganensis-like	533	OXA-181	/	/	2021	Rectal Swab	No	No
292H10	Klebsiella oxytoca	201	OXA-48	/	/	2021	Urine	No	No
294A3	Klebsiella oxytoca	2	NDM-1	CTX-M-15	CMY-4	2021	Rectal Swab	No	No
295B7	Klebsiella michiganensis	202	VIM-4	/	/	2021	Blood culture	No	No
298D9	Klebsiella michiganensis	135	KPC-2	/	/	2021	Rectal Swab	No	No
299B5	Klebsiella michiganensis-like	427	NDM-1	/	/	2021	Rectal Swab	No	No
301A7	Klebsiella michiganensis-like	310	VIM-1	/	/	2021	Rectal Swab	No	No
301C3	Klebsiella michiganensis	324	VIM-1	/	/	2021	Rectal Swab	No	No
304E6	Klebsiella michiganensis	254	NDM-1	SHV-12	/	2021	Rectal Swab	No	No
305A1	Klebsiella michiganensis	324	VIM-1	/	/	2021	Rectal Swab	No	No
305J6	Klebsiella michiganensis	819	NDM-1	SHV-12	/	2021	Rectal Swab	No	No
306J4	Klebsiella michiganensis	109-like	OXA-48	/	/	2021	Blood culture	No	No
308C2	Klebsiella michiganensis	381	NDM-1	CTX-M-15	/	2021	Rectal Swab	No	No
309H4	Klebsiella oxytoca	141	OXA-48	/	/	2021	Urine	No	No
311B4	Klebsiella michiganensis	324	VIM-4	/	/	2022	Rectal Swab	No	No
313D1	Klebsiella michiganensis	146	OXA-48	/	/	2022	Rectal Swab	No	No
314D2	Klebsiella michiganensis	50-like	OXA-48	/	/	2022	Rectal Swab	No	No
314F2	Klebsiella michiganensis-like	102	VIM-1	/	/	2022	Urine	No	No
314G7	Klebsiella michiganensis-like	310	VIM-1	/	/	2022	Rectal Swab	No	No
318H8	Klebsiella michiganensis	43	OXA-48	/	/	2022	Urine	No	No
319C4	Klebsiella michiganensis-like	83	OXA-48	CTX-M-15	DHA-1	2022	Rectal Swab	No	No

319E4	Klebsiella michiganensis	144	NDM-7	CTX-M-15	/	2022	Rectal Swab	No	No
322J8	Klebsiella oxytoca	2	OXA-48	/	/	2022	Rectal Swab	No	No
323A10	Klebsiella michiganensis	202	VIM-4	/	CMY-4	2022	Rectal Swab	No	No
325D1	Klebsiella michiganensis	324	VIM-4	/	/	2022	Rectal Swab	No	No
325J3	Klebsiella michiganensis-like	310	VIM-1	/	/	2022	Wound	No	No
327E10	Klebsiella grimontii	463	OXA-48	/	/	2022	Rectal Swab	No	No
327F10	Klebsiella michiganensis-like	310	VIM-1 + OXA-48	/	/	2022	Digestive biopsy	No	No
329D2	Klebsiella michiganensis	151	OXA-48	/	/	2022	Urine	No	No
329I9	Klebsiella michiganensis	44-like	KPC-3	/	/	2022	Bile	No	No
331D7	Klebsiella oxytoca	141	OXA-48	/	/	2022	Rectal Swab	No	No
333B10	Klebsiella michiganensis	151	OXA-48	/	/	2022	Urine	No	No
333J9	Klebsiella michiganensis	231	NDM-1	/	CMY-16	2022	Urine	No	No
335G6	Klebsiella michiganensis	503	OXA-48	/	/	2022	Rectal Swab	No	No
340F6	Klebsiella michiganensis	50	NDM-1 + VIM-1	CTX-M-15	/	2022	Rectal Swab	No	No
341B9	Klebsiella michiganensis	673	OXA-48	/	/	2022	Rectal Swab	No	No
341K6	Klebsiella michiganensis	177-like	VIM-1	/	/	2022	Rectal Swab	No	No
344B7	Klebsiella michiganensis	146	OXA-48	/	/	2022	Urine	No	No
344G10	Klebsiella michiganensis	27	OXA-48	/	DHA-1	2022	Rectal Swab	No	No
345B7	Klebsiella michiganensis-like	310	VIM-1	/	DHA-1	2022	Rectal Swab	No	No
346J2	Klebsiella michiganensis-like	310	VIM-1	/	/	2022	Rectal Swab	No	No
347G3	Klebsiella michiganensis-like	310	VIM-1	/	/	2022	Rectal Swab	No	No
348I8	Klebsiella oxytoca	2	OXA-48	CTX-M-15	/	2022	Rectal Swab	No	No
353A7	Klebsiella michiganensis	98	OXA-48	CTX-M-15	/	2022	Rectal Swab	No	No
355A2	Klebsiella michiganensis	144	NDM-7	CTX-M-15	/	2022	Rectal Swab	No	No
355F7	Klebsiella michiganensis	88	NDM-7	CTX-M-15	/	2022	Urine	No	No
358A3	Klebsiella michiganensis	85	OXA-48	/	/	2022	Rectal Swab	No	No
360C9	Klebsiella michiganensis	451-like	VIM-1	/	/	2022	Rectal Swab	No	No
360D4	Klebsiella michiganensis	324	VIM-4	/	/	2022	Rectal Swab	No	No
360D5	Klebsiella michiganensis	324	VIM-4	/	/	2022	Rectal Swab	No	No
360H8	Klebsiella pasteurii	416	OXA-48	CTX-M-37	/	2022	Rectal Swab	No	No
362B9	Klebsiella michiganensis-like	672	NDM-1	CTX-M-15	/	2023	Rectal Swab	No	No
363F1	Klebsiella michiganensis	144	NDM-7	CTX-M-3	/	2023	Rectal Swab	No	No
364A8	Klebsiella oxytoca	2	OXA-48	CTX-M-15	/	2023	Blood culture	No	No
366I2	Klebsiella oxytoca	21	OXA-48	/	/	2023	Rectal Swab	No	No
367B5	Klebsiella spallanzanii	876-like	OXA-48	CTX-M-15	/	2023	Rectal Swab	No	No
370D2	Klebsiella michiganensis-like	330	OXA-48	CTX-M-15, SHV-12	/	2023	Rectal Swab	No	No
371F6	Klebsiella michiganensis-like	310	VIM-1	/	/	2023	Urine	No	No
372H7	Klebsiella oxytoca	258-like	NDM-1	SHV-12	CMY-4	2023	Rectal Swab	No	No
374A2	Klebsiella michiganensis	108	VIM-1	/	/	2023	Rectal Swab	No	No
374A3	Klebsiella michiganensis	108	VIM-1	/	/	2023	Rectal Swab	No	No
375I4	Klebsiella michiganensis-like	671	NDM-7	/	/	2023	Rectal Swab	No	No
376J2	Klebsiella michiganensis	177-like	VIM-1	/	/	2023	Rectal Swab	No	No
377B3	Klebsiella michiganensis-like	310	VIM-1	/	/	2023	Rectal Swab	No	No
378A10	Klebsiella michiganensis	231	NDM-1	/	/	2023	Rectal Swab	No	No
379H9	Klebsiella michiganensis	144	NDM-1	CTX-M-15	/	2023	Rectal Swab	No	No
380D1	Klebsiella michiganensis-like	672	NDM-1	CTX-M-15	/	2023	Rectal Swab	No	No
380F5	Klebsiella michiganensis-like	82	NDM-1 + VIM-1	/	CMY-16	2023	Rectal Swab	No	No
380F6	Klebsiella michiganensis-like	310	VIM-1 + OXA-48	/	/	2023	Rectal Swab	No	No
380I4	Klebsiella michiganensis	202	OXA-48	CTX-M-15	/	2023	Urine	No	No
381I9	Klebsiella michiganensis	52	NDM-1	SHV-12	CMY-4	2023	Rectal Swab	No	No
379B9BIS	Klebsiella michiganensis-like	310	VIM-1 + OXA-48	CTX-M-15	/	2023	Rectal Swab	No	No
383A9	Klebsiella michiganensis	144	NDM-7	CTX-M-14	/	2023	Urine	No	No
385I10	Klebsiella michiganensis-like	310	VIM-1	/	DHA-1	2023	Undetermined	No	No
386F5	Klebsiella michiganensis	194	OXA-48	/	/	2023	Wound	No	No
387F8	Klebsiella michiganensis	50	OXA-181	CTX-M-15	/	2023	Other	No	No
391C3	Klebsiella michiganensis	88-like	OXA-48	/	/	2023	Urine	No	No
391C6	Klebsiella michiganensis	205	OXA-48	/	/	2023	Urine	No	No
393J5	Klebsiella michiganensis	144	NDM-1	/	/	2023	Rectal Swab	No	No
395C3	Klebsiella michiganensis	226	Non CPE	CTX-M-15	/	2023	Rectal Swab	No	No
395F8	Klebsiella michiganensis	144	NDM-1	/	/	2023	Rectal Swab	No	No
396G8	Klebsiella michiganensis	381	NDM-1	CTX-M-15	/	2023	Rectal Swab	No	No
396K6	Klebsiella michiganensis	144	NDM-1	/	/	2023	Rectal Swab	No	No
393B8BIS	Klebsiella grimontii	316-like	OXA-48	/	/	2023	Rectal Swab	No	No
398B2	Klebsiella michiganensis	27	Non CPE	/	DHA-1	2023	Rectal Swab	No	No
405G10	Klebsiella michiganensis	787	OXA-48	/	/	2023	Bone	No	No
408I3	Klebsiella michiganensis	151	NDM-1	/	/	2023	Rectal Swab	No	No
410F3	Klebsiella michiganensis	85	NDM-7	/	/	2023	Rectal Swab	No	No
411K9	Klebsiella michiganensis	85	OXA-48	/	/	2023	Urine	No	No
411K10	Klebsiella michiganensis	146	OXA-48	/	/	2023	Rectal Swab	No	No
412F8	Klebsiella michiganensis	308	OXA-48	CTX-M-15	/	2023	Urine	No	No
413B5	Klebsiella michiganensis	157	OXA-48	/	/	2023	Blood culture	No	No
414F10	Klebsiella michiganensis	202	NDM-7	/	/	2023	Rectal Swab	No	No
416C7	Klebsiella michiganensis	64	OXA-48	/	/	2023	Rectal Swab	No	No
416D3	Klebsiella michiganensis	623-like	NDM-7	CTX-M-8	/	2023	Blood culture	No	No
417C2	Klebsiella michiganensis	40	OXA-48	CTX-M-15	/	2023	Respiratory sample	No	No
102A10	Klebsiella oxytoca	176-like	Non CPE	CTX-M-15	/	2016	Urine	No	No
102B7	Klebsiella oxytoca	221	OXA-48	/	/	2016	Other	No	No
129E6	Klebsiella oxytoca	176-like	OXA-48	/	/	2017	Urine	No	No
128G1	Klebsiella oxytoca	176-like	OXA-48	/	/	2017	Urine	No	No
159E4	Klebsiella oxytoca	141	NDM-1	CTX-M-15	/	2017	Rectal Swab	No	No
160G5	Klebsiella oxytoca	2	OXA-48	CTX-M-15	/	2018	Rectal Swab	No	No
158A5	Klebsiella oxytoca	141	NDM-1	CTX-M-15	/	2017	Rectal Swab	No	No
161H4	Klebsiella oxytoca	2	OXA-48	CTX-M-15	/	2018	Blood culture	No	No
159A8	Klebsiella oxytoca	2	OXA-48	CTX-M-15	/	2017	Rectal Swab	No	No
165B10	Klebsiella oxytoca	2	OXA-48	CTX-M-15	/	2018	Urine	No	No
166B6	Klebsiella oxytoca	873-like	OXA-48	/	/	2018	Urine	No	No
166C2	Klebsiella oxytoca	141	OXA-48	/	/	2018	Urine	No	No
166E1	Klebsiella oxytoca	2	OXA-48	CTX-M-15	/	2018	Rectal Swab	No	No

166J1	Klebsiella oxytoca	141	Non CPE	CTX-M-15	/	2018	Urine	No	No
167G3	Klebsiella oxytoca	141	OXA-48	/	/	2018	Rectal Swab	No	No
167H6	Klebsiella oxytoca	2	OXA-48	CTX-M-15	/	2018	Urine	No	No
168C3	Klebsiella oxytoca	2	OXA-48	CTX-M-15	/	2018	Rectal Swab	No	No
168H10	Klebsiella oxytoca	2	OXA-48	/	/	2018	Rectal Swab	No	No
168I2	Klebsiella oxytoca	2	OXA-48	CTX-M-15	/	2018	Rectal Swab	No	No
169D5	Klebsiella oxytoca	141	Non CPE	CTX-M-15	/	2018	Urine	No	No
170E5	Klebsiella oxytoca	2	OXA-48	/	/	2018	Rectal Swab	No	No
173B5	Klebsiella oxytoca	141	OXA-48	SHV-12	/	2018	Rectal Swab	No	No
173C6	Klebsiella oxytoca	2	OXA-48	CTX-M-15	/	2018	Rectal Swab	No	No
173D1	Klebsiella oxytoca	2	OXA-48	CTX-M-15	/	2018	Rectal Swab	No	No
173J9	Klebsiella oxytoca	176-like	OXA-48	/	/	2018	Urine	No	No
174A2	Klebsiella oxytoca	2	OXA-48	CTX-M-15	/	2018	Rectal Swab	No	No
175F8	Klebsiella oxytoca	2	OXA-48	CTX-M-15	/	2018	Respiratory sample	No	No
176D10	Klebsiella oxytoca	2	OXA-48	CTX-M-15	/	2018	Rectal Swab	No	No
176G7	Klebsiella oxytoca	2	OXA-48	CTX-M-15	/	2018	Rectal Swab	No	No
176J3	Klebsiella oxytoca	2	OXA-48	CTX-M-15	/	2018	Blood culture	No	No
177D10	Klebsiella oxytoca	2	OXA-48	CTX-M-15	/	2018	Rectal Swab	No	No
178G10	Klebsiella oxytoca	2	OXA-48	CTX-M-15	/	2018	Undetermined	No	No
178H1	Klebsiella oxytoca	2	OXA-48	CTX-M-15	/	2018	Undetermined	No	No
178A9	Klebsiella oxytoca	2	OXA-48	CTX-M-15	/	2018	Urine	No	No
178B4	Klebsiella oxytoca	34	OXA-48	/	/	2018	Rectal Swab	No	No
178I9	Klebsiella oxytoca	2	OXA-48	CTX-M-15	/	2018	Rectal Swab	No	No
179B3	Klebsiella oxytoca	176-like	OXA-48	/	/	2018	Rectal Swab	No	No
179B4	Klebsiella oxytoca	176-like	OXA-48	/	/	2018	Urine	No	No
179C1	Klebsiella oxytoca	302	OXA-48	/	/	2018	Respiratory sample	No	No
182E7	Klebsiella oxytoca	2	OXA-48	CTX-M-15	/	2018	Rectal Swab	No	No
182J1	Klebsiella oxytoca	141	OXA-48	/	/	2018	Rectal Swab	No	No
183F9	Klebsiella oxytoca	199	OXA-48	/	/	2018	Rectal Swab	No	No
185H1	Klebsiella oxytoca	2	OXA-48	CTX-M-15	/	2018	Rectal Swab	No	No
186A10	Klebsiella oxytoca	199	OXA-48	/	/	2018	Undetermined	No	No
187E10	Klebsiella oxytoca	141	OXA-48	/	/	2018	Rectal Swab	No	No
189C9	Klebsiella oxytoca	2	OXA-48	/	/	2018	Urine	No	No
189F3	Klebsiella oxytoca	2	OXA-48	CTX-M-15	/	2018	Rectal Swab	No	No
189H8	Klebsiella oxytoca	2	OXA-48	CTX-M-15, TEM-24	/	2018	Rectal Swab	No	No
190B6	Klebsiella oxytoca	2	OXA-48	CTX-M-15	/	2018	Rectal Swab	No	No
190G3	Klebsiella oxytoca	2	OXA-48	CTX-M-15	/	2018	Rectal Swab	No	No
190I8	Klebsiella oxytoca	176	OXA-204	/	CMY-4, DHA-1	2018	Rectal Swab	No	No
191J6	Klebsiella oxytoca	245	OXA-48	/	/	2018	Wound	No	No
192A8	Klebsiella oxytoca	2	OXA-48	CTX-M-15	/	2018	Rectal Swab	No	No
192J7	Klebsiella oxytoca	2	OXA-48	/	/	2018	Rectal Swab	No	No
194D4	Klebsiella oxytoca	36	OXA-48	CTX-M-15	/	2018	Rectal Swab	No	No
198E10	Klebsiella oxytoca	141	OXA-48	/	/	2018	Other	No	No
199A8	Klebsiella oxytoca	2	OXA-48	CTX-M-15	/	2018	Rectal Swab	No	No
199B8	Klebsiella oxytoca	2	OXA-48	CTX-M-15	/	2018	Urine	No	No
200F2	Klebsiella oxytoca	2	OXA-48	CTX-M-15	/	2019	Rectal Swab	No	No
201E5	Klebsiella oxytoca	37	OXA-48	/	/	2019	Urine	No	No
201J8	Klebsiella oxytoca	2	OXA-48	CTX-M-15, TEM-24	/	2019	Rectal Swab	No	No
202A4	Klebsiella oxytoca	176	OXA-48	/	/	2019	Rectal Swab	No	No
203J7	Klebsiella oxytoca	176-like	OXA-48	/	/	2019	Other	No	No
205C7	Klebsiella oxytoca	2	OXA-48	CTX-M-15	/	2019	Rectal Swab	No	No
205J1	Klebsiella oxytoca	2	OXA-48	CTX-M-15	/	2019	Rectal Swab	No	No
209H3	Klebsiella oxytoca	2	OXA-48	CTX-M-15	/	2019	Rectal Swab	No	No
211H2	Klebsiella oxytoca	141	OXA-48	CTX-M-15	/	2019	Rectal Swab	No	No
213J3	Klebsiella oxytoca	2	OXA-48	CTX-M-15	/	2019	Rectal Swab	No	No
216F5	Klebsiella oxytoca	101	OXA-48	/	/	2019	Rectal Swab	No	No
216I10	Klebsiella oxytoca	2	OXA-48	CTX-M-15	/	2019	Urine	No	No
217A1	Klebsiella oxytoca	2	OXA-48	CTX-M-15	/	2019	Rectal Swab	No	No
217A8	Klebsiella oxytoca	2	OXA-48	CTX-M-15	/	2019	Rectal Swab	No	No
217A10	Klebsiella oxytoca	2	OXA-48	CTX-M-15	/	2019	Rectal Swab	No	No
218J3	Klebsiella oxytoca	2	OXA-48	CTX-M-15	/	2019	Rectal Swab	No	No
219B7	Klebsiella oxytoca	2	OXA-48	CTX-M-15	/	2019	Rectal Swab	No	No
219E10	Klebsiella oxytoca	2	OXA-48	CTX-M-15	/	2019	Rectal Swab	No	No
219H1	Klebsiella oxytoca	4-like	OXA-48	/	/	2019	Urine	No	No
221A7	Klebsiella oxytoca	2	OXA-48	CTX-M-15	/	2019	Urine	No	No
222A6	Klebsiella oxytoca	2	OXA-48	CTX-M-15, TEM-24	/	2019	Rectal Swab	No	No
222B9	Klebsiella oxytoca	141	OXA-48	/	/	2019	Urine	No	No
222E2	Klebsiella oxytoca	141	Non CPE	CTX-M-15	/	2019	Urine	No	No
226J3	Klebsiella oxytoca	2	OXA-48	CTX-M-15	/	2019	Rectal Swab	No	No
228D9	Klebsiella oxytoca	141	OXA-48	/	/	2019	Urine	No	No
229A8	Klebsiella oxytoca	2	OXA-48	CTX-M-15	/	2019	Rectal Swab	No	No
229B10	Klebsiella oxytoca	2	OXA-48	/	/	2019	Rectal Swab	No	No
229H10	Klebsiella oxytoca	2	OXA-48	/	/	2019	Rectal Swab	No	No
230J2	Klebsiella oxytoca	2	OXA-48	/	/	2019	Undetermined	No	No
231J3	Klebsiella oxytoca	2	OXA-48	CTX-M-15	/	2019	Urine	No	No
232F7	Klebsiella oxytoca	2	OXA-48	/	/	2019	Rectal Swab	No	No
233C10	Klebsiella oxytoca	2	OXA-48	/	/	2019	Urine	No	No
234B1	Klebsiella oxytoca	2	OXA-48	CTX-M-15	/	2019	Rectal Swab	No	No
236A3	Klebsiella oxytoca	2	OXA-48	CTX-M-15	/	2019	Rectal Swab	No	No
237E5	Klebsiella oxytoca	2	OXA-48	CTX-M-15	/	2019	Urine	No	No
238H3	Klebsiella oxytoca	2	OXA-48	/	/	2019	Urine	No	No
239J6	Klebsiella oxytoca	199	OXA-48	SHV-12	/	2019	Rectal Swab	No	No
240B3	Klebsiella oxytoca	2	OXA-48	CTX-M-15	/	2019	Rectal Swab	No	No
241D1	Klebsiella oxytoca	37	OXA-48	/	/	2019	Rectal Swab	No	No
241I7	Klebsiella oxytoca	141	OXA-48	CTX-M-15	/	2019	Urine	No	No
242F5	Klebsiella oxytoca	2	OXA-48	/	/	2019	Undetermined	No	No
242I8	Klebsiella oxytoca	141	OXA-48	/	/	2019	Rectal Swab	No	No
248G8	Klebsiella oxytoca	364	OXA-48	/	/	2020	Undetermined	No	No

250B9	Klebsiella oxytoca	2	OXA-48	CTX-M-15	/	2020	Rectal Swab	No	No
250G10	Klebsiella oxytoca	141	OXA-48	/	/	2020	Urine	Yes	Yes
251D6	Klebsiella oxytoca	2	OXA-48	CTX-M-15	/	2020	Rectal Swab	No	No
253E10	Klebsiella oxytoca	2	OXA-48	CTX-M-3	/	2020	Rectal Swab	Yes	Yes
256F6	Klebsiella oxytoca	2	OXA-48	CTX-M-15	/	2020	Rectal Swab	No	No
257J5	Klebsiella oxytoca	2	OXA-48	CTX-M-15	/	2020	Rectal Swab	No	No
259C9	Klebsiella oxytoca	2	OXA-48	CTX-M-15	/	2020	Rectal Swab	No	No
260E6	Klebsiella oxytoca	2	OXA-48	CTX-M-15	/	2020	Undetermined	No	No
262I2	Klebsiella oxytoca	37	OXA-48	/	/	2020	Rectal Swab	No	No
263G3	Klebsiella oxytoca	2	OXA-48	CTX-M-15	/	2020	Rectal Swab	No	No
263H7	Klebsiella oxytoca	141	OXA-48	/	/	2020	Urine	No	No
264A8	Klebsiella oxytoca	199	OXA-48	/	/	2020	Urine	No	No
266J9	Klebsiella oxytoca	176	OXA-48	CTX-M-15	/	2020	Rectal Swab	No	No
267B3	Klebsiella oxytoca	2	OXA-48	CTX-M-15	/	2020	Rectal Swab	No	No
268A2	Klebsiella oxytoca	2	OXA-48	CTX-M-15	/	2020	Urine	No	No
268G10	Klebsiella oxytoca	176	OXA-48	/	/	2020	Rectal Swab	No	No
270F6	Klebsiella oxytoca	2	OXA-48	CTX-M-15	/	2020	Urine	No	No
275G5	Klebsiella oxytoca	141	OXA-48	/	/	2020	Rectal Swab	No	No
275J6	Klebsiella oxytoca	605	OXA-48	/	/	2020	Urine	No	No
276F9	Klebsiella oxytoca	36	OXA-48	/	/	2020	Rectal Swab	No	No
133E9	Klebsiella oxytoca	2	OXA-48	CTX-M-15	/	2017	Urine	Yes	Yes
140D9	Klebsiella oxytoca	2	OXA-48	CTX-M-15	/	2017	Rectal Swab	Yes	Yes
277C4	Klebsiella oxytoca	36	NDM-1	SHV-12	CMY-4	2021	Rectal Swab	No	No
278G9	Klebsiella oxytoca	36	OXA-48	CTX-M-15	/	2021	Rectal Swab	No	No
278I1	Klebsiella oxytoca	176	OXA-48	/	/	2021	Urine	No	No
279F1	Klebsiella oxytoca	176	OXA-48	CTX-M-9	/	2021	Urine	No	No
279G8	Klebsiella oxytoca	141	OXA-48	/	/	2021	Undetermined	No	No
281D9	Klebsiella oxytoca	141	OXA-48	/	/	2021	Rectal Swab	No	No
282C7	Klebsiella oxytoca	2-like	Non CPE	CTX-M-15	/	2021	Rectal Swab	No	No
282G1	Klebsiella oxytoca	2	OXA-48	CTX-M-15	/	2021	Rectal Swab	No	No
283E3	Klebsiella oxytoca	2-like	OXA-48	CTX-M-15	/	2021	Rectal Swab	No	No
284E6	Klebsiella oxytoca	141	Non CPE	SHV-12	/	2021	Rectal Swab	No	No
284H2	Klebsiella oxytoca	2	OXA-48	CTX-M-15	/	2021	Urine	No	No
285B6	Klebsiella oxytoca	141	OXA-48	/	/	2021	Blood culture	No	No
285A2	Klebsiella oxytoca	199	Non CPE	SHV-12	/	2021	Urine	No	No
285F4	Klebsiella oxytoca	176-like	OXA-48	/	/	2021	Other	No	No
287E10	Klebsiella oxytoca	65	OXA-48	/	/	2021	Urine	No	No
289J7	Klebsiella oxytoca	2	OXA-48	CTX-M-15	/	2021	Rectal Swab	No	No
291A9	Klebsiella oxytoca	141	OXA-48	TEM-32	/	2021	Other	No	No
292I5	Klebsiella oxytoca	176	OXA-48	/	/	2021	Rectal Swab	No	No
293C8	Klebsiella oxytoca	2	OXA-48	CTX-M-15	/	2021	Urine	No	No
295B8	Klebsiella oxytoca	141	OXA-48	/	/	2021	Rectal Swab	No	No
295G5	Klebsiella oxytoca	176	OXA-48	/	/	2021	Rectal Swab	No	No
299E8	Klebsiella oxytoca	2	OXA-48	CTX-M-15	/	2021	Rectal Swab	No	No
299I3	Klebsiella oxytoca	36	VIM-1	/	/	2021	Blood culture	No	No
301D8	Klebsiella oxytoca	2	OXA-48	CTX-M-15	/	2021	Urine	No	No
301I2	Klebsiella oxytoca	2	OXA-48	CTX-M-15	/	2021	Rectal Swab	No	No
284D8TER	Klebsiella oxytoca	141	NDM-1	CTX-M-15	/	2021	Rectal Swab	No	No
302C4	Klebsiella oxytoca	302	OXA-48	/	/	2021	Rectal Swab	No	No
304A2	Klebsiella oxytoca	2	OXA-48	CTX-M-15	/	2021	Rectal Swab	No	No
307D9	Klebsiella oxytoca	2	OXA-48	CTX-M-15	/	2021	Rectal Swab	No	No
310D3	Klebsiella oxytoca	2	NDM-1	CTX-M-15	/	2021	Urine	No	No
311G4	Klebsiella oxytoca	2	OXA-48	/	/	2022	Rectal Swab	No	No
313B10	Klebsiella oxytoca	2	OXA-48	CTX-M-15	/	2022	Rectal Swab	No	No
313I1	Klebsiella oxytoca	2	OXA-48	CTX-M-15	/	2022	Rectal Swab	No	No
314G2	Klebsiella oxytoca	141	OXA-48	/	/	2022	Rectal Swab	No	No
315H10	Klebsiella oxytoca	141	OXA-48	/	/	2022	Urine	No	No
315J7	Klebsiella oxytoca	2	OXA-48	CTX-M-15	/	2022	Urine	No	No
316E5	Klebsiella oxytoca	141	OXA-48	/	/	2022	Rectal Swab	No	No
316J10	Klebsiella oxytoca	141	OXA-48	CTX-M-15	/	2022	Urine	No	No
318D1	Klebsiella oxytoca	2	OXA-48	CTX-M-15	/	2022	Urine	No	No
318D6	Klebsiella oxytoca	2	OXA-48	CTX-M-15	/	2022	Rectal Swab	No	No
320B5	Klebsiella oxytoca	761	OXA-48	/	/	2022	Wound	No	No
320I1	Klebsiella oxytoca	2	OXA-48	CTX-M-15	/	2022	Rectal Swab	No	No
321G6	Klebsiella oxytoca	141	OXA-48	CTX-M-15	/	2022	Urine	No	No
323E8	Klebsiella oxytoca	2	NDM-1	/	CMY-6	2022	Rectal Swab	No	No
325B3	Klebsiella oxytoca	2	OXA-48	/	/	2022	Respiratory sample	No	No
325C7	Klebsiella oxytoca	364	OXA-48	/	/	2022	Rectal Swab	No	No
326G5	Klebsiella oxytoca	2	OXA-48	CTX-M-15	/	2022	Rectal Swab	No	No
326I1	Klebsiella oxytoca	141	OXA-48	/	/	2022	Rectal Swab	No	No
327G3	Klebsiella oxytoca	141	OXA-48	/	/	2022	Rectal Swab	No	No
327H10	Klebsiella oxytoca	2	OXA-48	/	/	2022	Rectal Swab	No	No
328D9	Klebsiella oxytoca	2	OXA-48	CTX-M-15	/	2022	Rectal Swab	No	No
328J4	Klebsiella oxytoca	2	OXA-48	CTX-M-15	/	2022	Rectal Swab	No	No
329F5	Klebsiella oxytoca	699	OXA-48	/	/	2022	Ascite	No	No
330B1	Klebsiella oxytoca	2	OXA-48	CTX-M-15, SHV-12	DHA-1	2022	Rectal Swab	No	No
331J5	Klebsiella oxytoca	2	OXA-48	CTX-M-15	/	2022	Urine	No	No
332F2	Klebsiella oxytoca	2	OXA-48	CTX-M-15	/	2022	Rectal Swab	No	No
332G10	Klebsiella oxytoca	2	OXA-48	CTX-M-15	/	2022	Rectal Swab	No	No
332J6	Klebsiella oxytoca	2	OXA-48	CTX-M-15	/	2022	Bile	No	No
333B3	Klebsiella oxytoca	176	OXA-48	/	/	2022	Urine	No	No
333D5	Klebsiella oxytoca	2	OXA-48	CTX-M-15	/	2022	Urine	No	No
333I2	Klebsiella oxytoca	2	OXA-181	CTX-M-15	/	2022	Urine	No	No
335K3	Klebsiella oxytoca	2	OXA-48	CTX-M-15	/	2022	Urine	No	No
336J1	Klebsiella oxytoca	2	OXA-48	/	/	2022	Urine	No	No
336J2	Klebsiella oxytoca	2	OXA-48	CTX-M-15	/	2022	Urine	No	No
339A10	Klebsiella oxytoca	141	OXA-48	CTX-M-15	/	2022	Rectal Swab	No	No
339J2	Klebsiella oxytoca	605	OXA-48	/	/	2022	Rectal Swab	No	No

340A7	Klebsiella oxytoca	141	OXA-48	/	/	2022	Bile	No	No
342B3	Klebsiella oxytoca	141	OXA-48	CTX-M-15	/	2022	Other	No	No
342F4	Klebsiella oxytoca	65	OXA-48	/	/	2022	Urine	No	No
345A6	Klebsiella oxytoca	2	OXA-48	CTX-M-15	/	2022	Urine	No	No
346G5	Klebsiella oxytoca	2	OXA-48	/	/	2022	Rectal Swab	No	No
346J9	Klebsiella oxytoca	2	OXA-48	CTX-M-15	/	2022	Urine	No	No
347C9	Klebsiella oxytoca	2	OXA-48	/	/	2022	Rectal Swab	No	No
347D2	Klebsiella oxytoca	65	OXA-48	/	/	2022	Urine	No	No
347I6	Klebsiella oxytoca	176	VIM-4	/	/	2022	Blood culture	No	No
348B2	Klebsiella oxytoca	2	OXA-48	CTX-M-15	/	2022	Other	No	No
349D6	Klebsiella oxytoca	2	OXA-48	/	/	2022	Urine	No	No
350A7	Klebsiella oxytoca	141	OXA-48	/	/	2022	Rectal Swab	No	No
350B7	Klebsiella oxytoca	2	OXA-48	CTX-M-15	/	2022	Rectal Swab	No	No
350C10	Klebsiella oxytoca	2	OXA-48	CTX-M-15	/	2022	Rectal Swab	No	No
351J6	Klebsiella oxytoca	37	VIM-1	SHV-12	/	2022	Rectal Swab	No	No
351J10	Klebsiella oxytoca	2	OXA-48	CTX-M-15	/	2022	Rectal Swab	No	No
352H2	Klebsiella oxytoca	141	OXA-48	/	/	2022	Rectal Swab	No	No
353F2	Klebsiella oxytoca	141	OXA-48	/	/	2022	Rectal Swab	No	No
353I10	Klebsiella oxytoca	302	OXA-48	/	/	2022	Urine	No	No
353K2	Klebsiella oxytoca	141	OXA-48	/	/	2022	Rectal Swab	No	No
354B1	Klebsiella oxytoca	2	OXA-48	CTX-M-15	/	2022	Rectal Swab	No	No
354H5	Klebsiella oxytoca	2	OXA-48	CTX-M-15	/	2022	Rectal Swab	No	No
355J3	Klebsiella oxytoca	2	OXA-48	/	/	2022	Rectal Swab	No	No
356C6	Klebsiella oxytoca	141	Non CPE	CTX-M-15	/	2022	Rectal Swab	No	No
356J9	Klebsiella oxytoca	1	OXA-181	CTX-M-15	/	2022	Rectal Swab	No	No
357D3	Klebsiella oxytoca	2	NDM-1 + OXA-48	CTX-M-15	CMY-4	2022	Rectal Swab	No	No
358C5	Klebsiella oxytoca	2	VIM-4	CTX-M-15	/	2022	Rectal Swab	No	No
358D10	Klebsiella oxytoca	176-like	OXA-48	/	/	2022	Respiratory sample	No	No
360F10	Klebsiella oxytoca	176	OXA-48	/	/	2022	Rectal Swab	No	No
360H3	Klebsiella oxytoca	2	OXA-48	CTX-M-15	/	2022	Rectal Swab	No	No
360H6	Klebsiella oxytoca	2	OXA-48	CTX-M-15	/	2022	Rectal Swab	No	No
365C2	Klebsiella oxytoca	176	VIM-4 + OXA-48	/	/	2023	Bone	No	No
365H10	Klebsiella oxytoca	2	NDM-1	/	CMY-6	2023	Rectal Swab	No	No
365J9	Klebsiella oxytoca	141	OXA-48	CTX-M-15	/	2023	Rectal Swab	No	No
364A5	Klebsiella oxytoca	302	OXA-48	/	/	2023	Urine	No	No
368H7	Klebsiella oxytoca	2	OXA-48	/	/	2023	Urine	No	No
369G3	Klebsiella oxytoca	2	OXA-48	/	/	2023	Urine	No	No
369I6	Klebsiella oxytoca	286-like	OXA-48	CTX-M-15	/	2023	Urine	No	No
370B5	Klebsiella oxytoca	2	OXA-48	CTX-M-15	/	2023	Blood culture	No	No
370D10	Klebsiella oxytoca	199	NDM-1	CTX-M-15, CTX-M-	/	2023	Rectal Swab	No	No
370H8	Klebsiella oxytoca	2	OXA-48	/	/	2023	Rectal Swab	No	No
371C5	Klebsiella oxytoca	199	OXA-48	/	/	2023	Bone	No	No
371I4	Klebsiella oxytoca	2	OXA-48	CTX-M-15	/	2023	Urine	No	No
372B9	Klebsiella oxytoca	2	VIM-4	CTX-M-15	/	2023	Urine	No	No
373B2	Klebsiella oxytoca	141	Non CPE	/	DHA-1	2023	Urine	No	No
373B9	Klebsiella oxytoca	2	OXA-48	CTX-M-15	/	2023	Rectal Swab	No	No
373C3	Klebsiella oxytoca	729	OXA-48	/	/	2023	Urine	No	No
374H10	Klebsiella oxytoca	141	OXA-48	CTX-M-15	/	2023	Urine	No	No
374K8	Klebsiella oxytoca	141	OXA-48	CTX-M-15	/	2023	Urine	No	No
376C4	Klebsiella oxytoca	141	OXA-48	/	/	2023	Urine	No	No
376K6	Klebsiella oxytoca	601	NDM-1	/	/	2023	Rectal Swab	No	No
377F10	Klebsiella oxytoca	2	OXA-48	CTX-M-15	/	2023	Rectal Swab	No	No
377G7	Klebsiella oxytoca	176	NDM-5	/	/	2023	Rectal Swab	No	No
378B5	Klebsiella oxytoca	141	OXA-48	CTX-M-15	/	2023	Blood culture	No	No
378C9	Klebsiella oxytoca	141	OXA-48	/	/	2023	Rectal Swab	No	No
380J1	Klebsiella oxytoca	199	OXA-48	/	/	2023	Bone	No	No
381H2	Klebsiella oxytoca	141	OXA-48	/	/	2023	Rectal Swab	No	No
381H7	Klebsiella oxytoca	161-like	NDM-1	SHV-12	/	2023	Rectal Swab	No	No
381I3	Klebsiella oxytoca	2	OXA-48	/	/	2023	Urine	No	No
383B4	Klebsiella oxytoca	36	OXA-48	/	/	2023	Urine	No	No
383H8	Klebsiella oxytoca	2	OXA-48	CTX-M-15	/	2023	Urine	No	No
385B7	Klebsiella oxytoca	2	OXA-48	CTX-M-15	/	2023	Rectal Swab	No	No
386A7	Klebsiella oxytoca	36	KPC-3	/	/	2023	Rectal Swab	No	No
386I2	Klebsiella oxytoca	141	Non CPE	SHV-12	/	2023	Rectal Swab	No	No
387B5	Klebsiella oxytoca	2	OXA-48	CTX-M-15	/	2023	Rectal Swab	No	No
387H6	Klebsiella oxytoca	176	VIM-1	M-15, SHV-12, CTX	/	2023	Rectal Swab	No	No
388G2	Klebsiella oxytoca	2	OXA-48	CTX-M-15	/	2023	Rectal Swab	No	No
388H4	Klebsiella oxytoca	2	OXA-48	CTX-M-15	/	2023	Rectal Swab	No	No
389G2	Klebsiella oxytoca	101	OXA-48	/	/	2023	Rectal Swab	No	No
390I5	Klebsiella oxytoca	199	OXA-48	/	/	2023	Urine	No	No
393H3	Klebsiella oxytoca	141	OXA-48	/	/	2023	Urine	No	No
393I10	Klebsiella oxytoca	2	OXA-48	/	DHA-1	2023	Rectal Swab	No	No
394B2	Klebsiella oxytoca	2	OXA-48	/	/	2023	Bone	No	No
394K3	Klebsiella oxytoca	364	Non CPE	/	DHA-1	2023	Urine	No	No
394K4	Klebsiella oxytoca	37	NDM-1	/	CMY-4	2023	Rectal Swab	No	No
394K6	Klebsiella oxytoca	37	NDM-1	/	CMY-4	2023	Rectal Swab	No	No
395K7	Klebsiella oxytoca	2	OXA-48	CTX-M-15	/	2023	Rectal Swab	No	No
395K5	Klebsiella oxytoca	2	OXA-48	CTX-M-15	/	2023	Rectal Swab	No	No
396J5	Klebsiella oxytoca	141	OXA-48	/	/	2023	Rectal Swab	No	No
397C7	Klebsiella oxytoca	176-like	OXA-48	/	/	2023	Urine	No	No
402G7	Klebsiella oxytoca	796	OXA-48	/	/	2023	Urine	No	No
403A7	Klebsiella oxytoca	796	OXA-48	/	/	2023	Rectal Swab	No	No
403G2	Klebsiella oxytoca	65	OXA-48	/	/	2023	Rectal Swab	No	No
406B4	Klebsiella oxytoca	199	NDM-1	SHV-12	CMY-4	2023	Urine	No	No
404B10	Klebsiella oxytoca	37	NDM-1	/	CMY-4	2023	Rectal Swab	No	No
407A9	Klebsiella oxytoca	2	OXA-48	CTX-M-15	/	2023	Urine	No	No
407H8	Klebsiella oxytoca	176	OXA-48	/	/	2023	Rectal Swab	No	No
409H10	Klebsiella oxytoca	20	NDM-1	CTX-M-15	/	2023	Rectal Swab	No	No

410H10	Klebsiella oxytoca	37	NDM-1	/	CMY-4	2023	Rectal Swab	No	No
410K7	Klebsiella oxytoca	2	OXA-48	CTX-M-15	/	2023	Rectal Swab	No	No
411H9	Klebsiella oxytoca	2	OXA-48	CTX-M-15	/	2023	Rectal Swab	No	No
412H9	Klebsiella oxytoca	796	OXA-48	/	/	2023	Rectal Swab	No	No
415H4	Klebsiella oxytoca	37	NDM-1	/	CMY-4	2023	Rectal Swab	No	No
415H5	Klebsiella oxytoca	37	NDM-1	/	CMY-4	2023	Rectal Swab	No	No
416H5	Klebsiella oxytoca	2	OXA-48	CTX-M-15	/	2023	Urine	No	No
419A3	Klebsiella oxytoca	141	OXA-48	/	/	2023	Rectal Swab	No	No
419A8	Klebsiella oxytoca	141	OXA-48	/	/	2023	Rectal Swab	No	No
420C7	Klebsiella oxytoca	364	OXA-48	CTX-M-15	/	2023	Blood culture	No	No
419A1	Klebsiella oxytoca	141	OXA-48	/	/	2023	Rectal Swab	No	No
113C6	Klebsiella oxytoca	141	OXA-48	/	/	2016	Urine	No	No
183D1	Klebsiella oxytoca	2	OXA-48	CTX-M-15	/	2018	Rectal Swab	No	No
269F5	Klebsiella oxytoca	98	OXA-48	/	/	2020	Urine	No	No
197H2	Klebsiella oxytoca	141	Non CPE	CTX-M-15	/	2018	Undetermined	No	No
331B4	Klebsiella oxytoca	2	OXA-48	CTX-M-15	/	2022	Rectal Swab	No	No
380F7	Klebsiella oxytoca	141	OXA-48	/	/	2023	Rectal Swab	No	No
259B1	Klebsiella oxytoca	2	NDM-1	/	/	2020	Rectal Swab	No	No
390F7	Klebsiella oxytoca	2	OXA-48	CTX-M-15	/	2023	Rectal Swab	No	No
292G2	Klebsiella oxytoca	2	NDM-1	/	DHA-1	2021	Rectal Swab	No	No
323A7	Klebsiella oxytoca	2	NDM-1	/	CMY-6	2022	Rectal Swab	No	No
413A8	Klebsiella oxytoca	2	OXA-48	CTX-M-15	/	2023	Rectal Swab	No	No
294J5Bis	Klebsiella oxytoca	218	OXA-48	CTX-M-15	/	2021	Rectal Swab	No	No
