# Supplementary material for: Carbapenemase-producing Klebsiella oxytoca complex in France (2017–2023): molecular epidemiology integrated with virulence and biofilm phenotypes
Source: Emerg Microbes Infect. 2026 Aug 3;15(1):2713319. doi: 10.1080/22221751.2026.2713319 (PMC13508490; doi:10.1080/22221751.2026.2713319)
Supplement: Supplementary file 4 [file TEMI_A_2713319_SM6354.pdf]

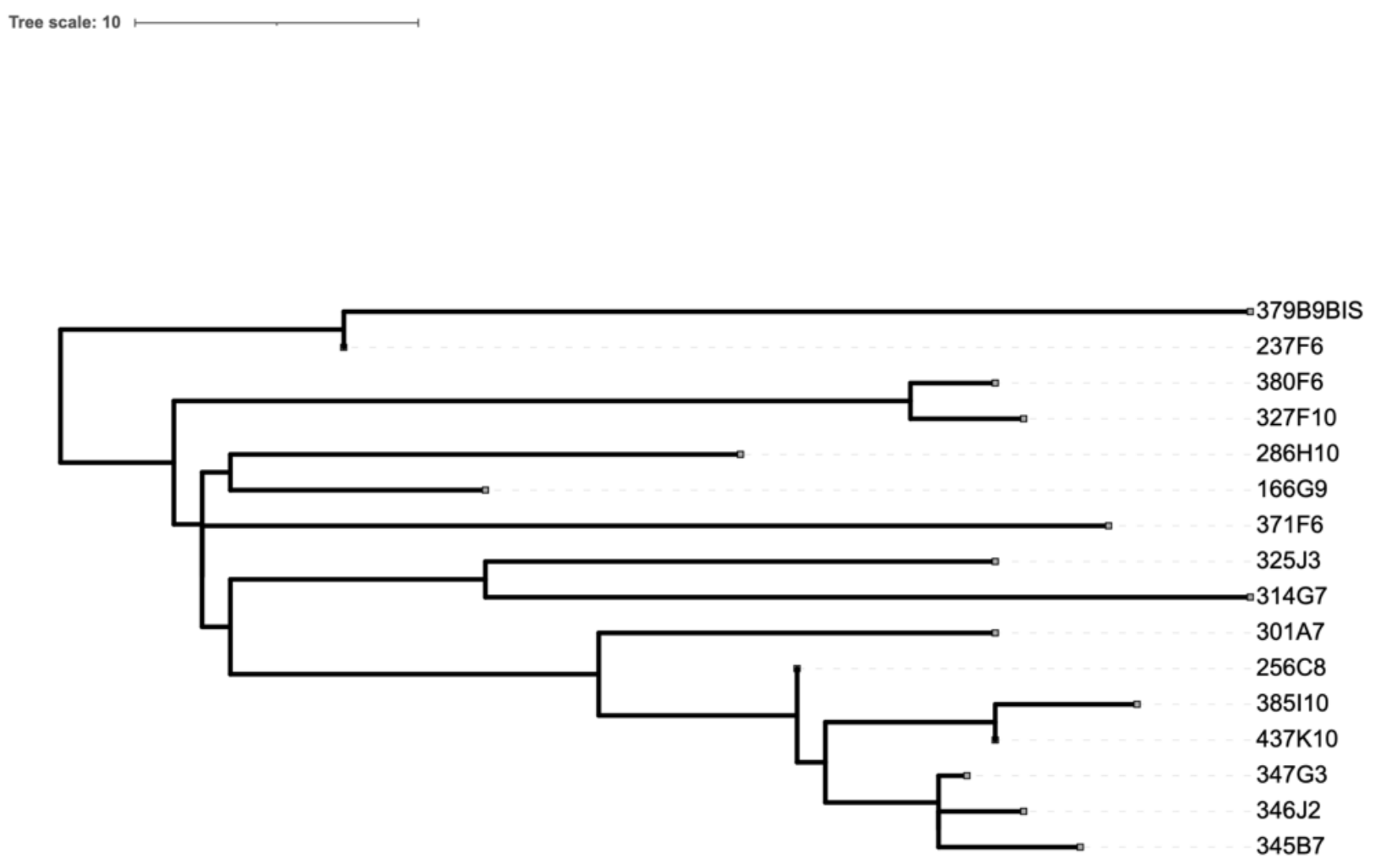

Isolate	Region	379B9BIS	237F6	380F6	327F10	286H10	166G9	371F6	325J3	314G7	301A7	256C8	385I10	437K10	347G3	346J2	345B7
379B9BIS	CHU - LYON - CROIX ROUSSE	0	32	75	76	66	57	79	75	84	75	68	80	75	74	76	78
237F6	CHU - LYON - CROIX ROUSSE	32	0	43	44	34	25	47	43	52	43	36	48	43	42	44	46
380F6	CHU - LYON - CROIX ROUSSE	75	43	0	7	49	40	62	58	67	58	51	63	58	57	59	61
327F10	CHU - LYON - CROIX ROUSSE	76	44	7	0	50	41	63	59	68	59	52	64	59	58	60	62
286H10	CHU - LYON - CROIX ROUSSE	66	34	49	50	0	27	51	47	56	47	40	52	47	46	48	50
166G9	CHU - LYON - CROIX ROUSSE	57	25	40	41	27	0	42	38	47	38	31	43	38	37	39	41
371F6	CHU - LYON - CROIX ROUSSE	79	47	62	63	51	42	0	60	69	60	53	65	60	59	61	63
325J3	LAM UNILIANS DECINES	75	43	58	59	47	38	60	0	45	54	47	59	54	53	55	57
314G7	CHU - LYON - CROIX ROUSSE	84	52	67	68	56	47	69	45	0	63	56	68	63	62	64	66
301A7	CHU - LYON - CROIX ROUSSE	75	43	58	59	47	38	60	54	63	0	21	33	28	27	29	31
256C8	CHU - LYON - CROIX ROUSSE	68	36	51	52	40	31	53	47	56	21	0	12	7	6	8	10
385I10	CHU - LYON - CROIX ROUSSE	80	48	63	64	52	43	65	59	68	33	12	0	5	16	18	20
437K10	CHU - LYON - CROIX ROUSSE	75	43	58	59	47	38	60	54	63	28	7	5	0	11	13	15
347G3	CHU - LYON - CROIX ROUSSE	74	42	57	58	46	37	59	53	62	27	6	16	11	0	4	6
346J2	CHU - LYON - CROIX ROUSSE	76	44	59	60	48	39	61	55	64	29	8	18	13	4	0	8
345B7	CHU - LYON - CROIX ROUSSE	78	46	61	62	50	41	63	57	66	31	10	20	15	6	8	0

SNP: 0, 8, 16, 25, 33, 42, 50, 58, 67, 75, 84
