# Supplementary material for: Carbapenemase-producing Klebsiella oxytoca complex in France (2017–2023): molecular epidemiology integrated with virulence and biofilm phenotypes
Source: Emerg Microbes Infect. 2026 Aug 3;15(1):2713319. doi: 10.1080/22221751.2026.2713319 (PMC13508490; doi:10.1080/22221751.2026.2713319)
Supplement: Supplementary file 5 [file TEMI_A_2713319_SM6355.pdf]

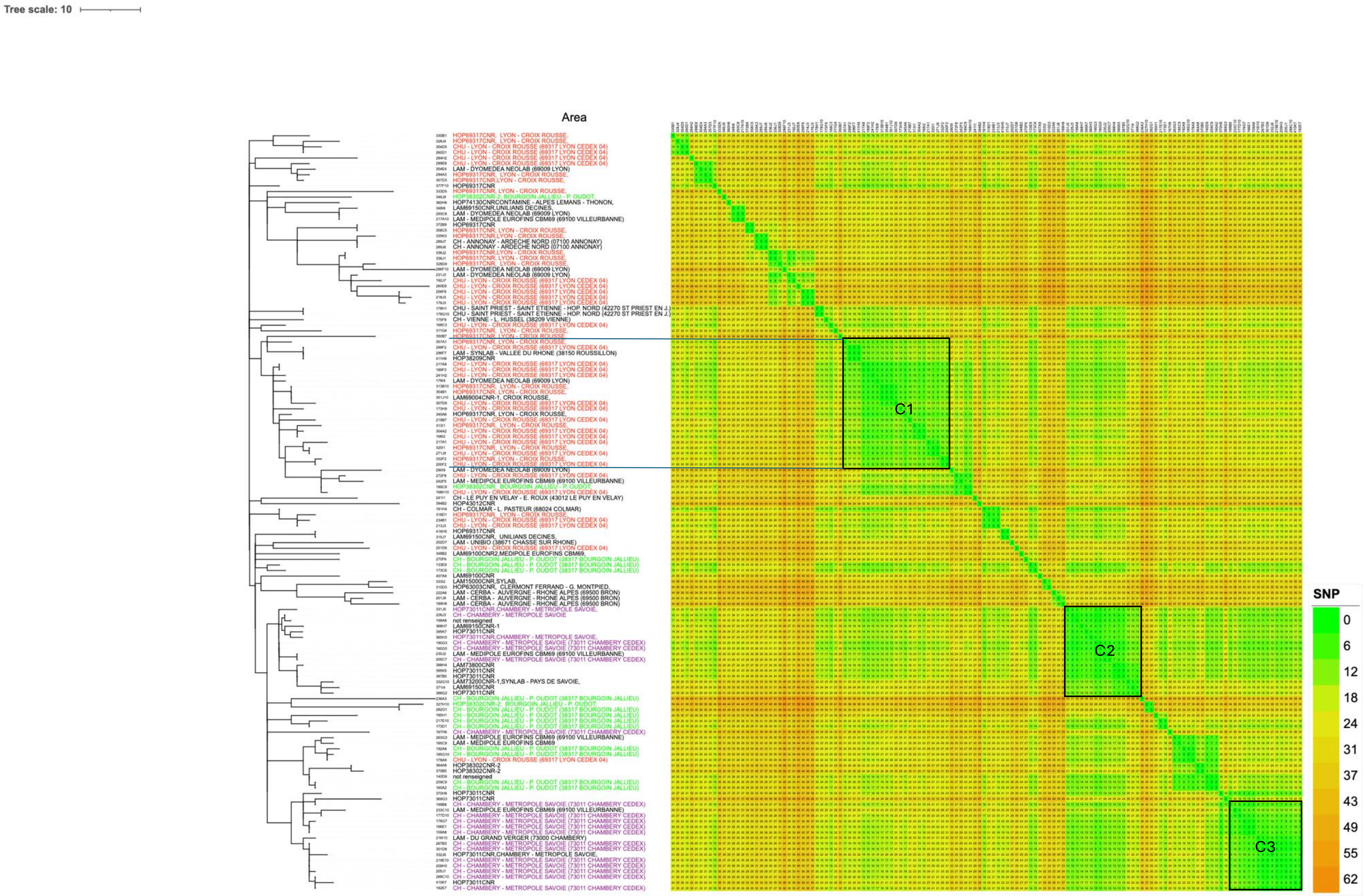

Supplementary Data1. *Klebsiella oxytoca* –ST2-Auvergne Rhône Alpes- Reference strain: 160A2
