# Supplementary material for: Carbapenemase-producing Klebsiella oxytoca complex in France (2017–2023): molecular epidemiology integrated with virulence and biofilm phenotypes
Source: Emerg Microbes Infect. 2026 Aug 3;15(1):2713319. doi: 10.1080/22221751.2026.2713319 (PMC13508490; doi:10.1080/22221751.2026.2713319)
Supplement: Supplementary file 6 [file TEMI_A_2713319_SM6356.pdf]

Gene	Category	ST-2 (n=184)		ST-141 (n=71)		Other ST (n=374)	
		n	%	n	%	n	%
entA	Entérobactine	178	96.7%	68	95.8%	374	98.7%
entB	Entérobactine	178	96.7%	68	95.8%	374	98.7%
entC	Entérobactine	178	96.7%	68	95.8%	373	98.4%
entD	Entérobactine	0	0.0%	0	0.0%	1	0.3%
entE	Entérobactine	178	96.7%	68	95.8%	374	98.7%
entF	Entérobactine	178	96.7%	68	95.8%	374	98.7%
entS	Entérobactine	178	96.7%	68	95.8%	374	98.7%
fepA	Entérobactine	177	96.2%	68	95.8%	374	98.7%
fepB	Entérobactine	178	96.7%	68	95.8%	374	98.7%
fepC	Entérobactine	178	96.7%	68	95.8%	374	98.7%
fepD	Entérobactine	178	96.7%	68	95.8%	374	98.7%
fepG	Entérobactine	178	96.7%	68	95.8%	374	98.7%
fes	Entérobactine	0	0.0%	0	0.0%	1	0.3%
ybtA	Yersiniabactine	176	95.7%	68	95.8%	300	79.2%
ybtE	Yersiniabactine	176	95.7%	68	95.8%	300	79.2%
ybtP	Yersiniabactine	176	95.7%	68	95.8%	299	78.9%
ybtQ	Yersiniabactine	176	95.7%	68	95.8%	302	79.7%
ybtS	Yersiniabactine	176	95.7%	68	95.8%	302	79.7%
ybtT	Yersiniabactine	176	95.7%	68	95.8%	299	78.9%
ybtU	Yersiniabactine	176	95.7%	68	95.8%	299	78.9%
ybtX	Yersiniabactine	176	95.7%	68	95.8%	302	79.7%
irp1	Yersiniabactine	176	95.7%	68	95.8%	298	78.6%
irp2	Yersiniabactine	173	94.0%	67	94.4%	299	78.9%
fyuA	Yersiniabactine	176	95.7%	68	95.8%	302	79.7%
iroB	Salmochelin	0	0.0%	61	85.9%	41	10.8%
iroC	Salmochelin	0	0.0%	0	0.0%	1	0.3%
iroD	Salmochelin	0	0.0%	0	0.0%	2	0.5%
iroE	Salmochelin	0	0.0%	0	0.0%	2	0.5%
iroN	Salmochelin	0	0.0%	2	2.8%	1	0.3%
iucA	Aérobactine	0	0.0%	0	0.0%	1	0.3%
iucB	Aérobactine	0	0.0%	0	0.0%	1	0.3%
iucC	Aérobactine	0	0.0%	0	0.0%	1	0.3%
iucD	Aérobactine	0	0.0%	0	0.0%	1	0.3%
iutA	Aérobactine	176	95.7%	68	95.8%	375	98.9%
chuU	Hème	0	0.0%	0	0.0%	1	0.3%
chuV	Hème	0	0.0%	0	0.0%	1	0.3%
chuW	Hème	0	0.0%	0	0.0%	3	0.8%
chuX	Hème	0	0.0%	0	0.0%	3	0.8%
chuY	Hème	0	0.0%	0	0.0%	3	0.8%
shuU	Hème	0	0.0%	0	0.0%	2	0.5%
shuV	Hème	0	0.0%	0	0.0%	2	0.5%
hmoX	Hème	171	92.9%	1	1.4%	172	45.4%
sitA	Fer-autres	178	96.7%	68	95.8%	374	98.7%
sitB	Fer-autres	178	96.7%	68	95.8%	374	98.7%
sitC	Fer-autres	178	96.7%	68	95.8%	374	98.7%
sitD	Fer-autres	0	0.0%	0	0.0%	2	0.5%
fimA	Fimbriae-1	175	95.1%	68	95.8%	306	80.7%
fimB	Fimbriae-1	175	95.1%	68	95.8%	302	79.7%
fimC	Fimbriae-1	178	96.7%	68	95.8%	374	98.7%
fimD	Fimbriae-1	171	92.9%	62	87.3%	333	87.9%
fimE	Fimbriae-1	176	95.7%	68	95.8%	309	81.5%
fimF	Fimbriae-1	178	96.7%	67	94.4%	364	96.0%
fimG	Fimbriae-1	178	96.7%	68	95.8%	374	98.7%
fimH	Fimbriae-1	178	96.7%	68	95.8%	374	98.7%
fimI	Fimbriae-1	178	96.7%	68	95.8%	372	98.2%
mrkA	Fimbriae-3	176	95.7%	68	95.8%	373	98.4%
mrkB	Fimbriae-3	176	95.7%	68	95.8%	373	98.4%
mrkC	Fimbriae-3	176	95.7%	68	95.8%	374	98.7%
mrkD	Fimbriae-3	2	1.1%	0	0.0%	4	1.1%
mrkF	Fimbriae-3	178	96.7%	68	95.8%	374	98.7%
mrkH	Fimbriae-3	178	96.7%	68	95.8%	372	98.2%
mrkJ	Fimbriae-3	177	96.2%	67	94.4%	371	97.9%
ecpA	ECP	0	0.0%	0	0.0%	1	0.3%
ecpC	ECP	178	96.7%	68	95.8%	373	98.4%
ecpD	ECP	178	96.7%	68	95.8%	373	98.4%
ecpE	ECP	0	0.0%	0	0.0%	1	0.3%
yagY--ecpB	ECP	178	96.7%	68	95.8%	372	98.2%
yagZ--ecpA	ECP	178	96.7%	68	95.8%	375	98.9%
ykgK--ecpR	ECP	173	94.0%	63	88.7%	361	95.3%
papB	P-fimbriae	0	0.0%	0	0.0%	1	0.3%
papC	P-fimbriae	0	0.0%	0	0.0%	1	0.3%
papD	P-fimbriae	0	0.0%	0	0.0%	1	0.3%
papE	P-fimbriae	0	0.0%	0	0.0%	1	0.3%
papG-III	P-fimbriae	0	0.0%	0	0.0%	1	0.3%
papH	P-fimbriae	0	0.0%	0	0.0%	1	0.3%
papI	P-fimbriae	0	0.0%	0	0.0%	1	0.3%
papJ	P-fimbriae	0	0.0%	0	0.0%	1	0.3%
papK	P-fimbriae	0	0.0%	0	0.0%	1	0.3%
papX	P-fimbriae	0	0.0%	0	0.0%	1	0.3%
sfaD	S-fimbriae	0	0.0%	0	0.0%	1	0.3%
sfaE	S-fimbriae	0	0.0%	0	0.0%	1	0.3%
sfaG	S-fimbriae	0	0.0%	0	0.0%	1	0.3%
sfaY	S-fimbriae	0	0.0%	0	0.0%	1	0.3%
pilN	Pili-IV	0	0.0%	1	1.4%	0	0.0%
pilX	Pili-IV	0	0.0%	0	0.0%	1	0.3%
ppdD	Pili-IV	178	96.7%	68	95.8%	376	99.2%
tcpC	Pili-IV	0	0.0%	0	0.0%	2	0.5%
hek	Adhésines	0	0.0%	0	0.0%	1	0.3%
tsh	Adhésines	0	0.0%	0	0.0%	1	0.3%
ompA	Adhésines	178	96.7%	68	95.8%	375	98.9%
rcsA	Capsule	178	96.7%	68	95.8%	374	98.7%
rcsB	Capsule	177	96.2%	68	95.8%	374	98.7%
